# The Min System and Nucleoid Occlusion Are Not Required for Identifying the Division Site in *Bacillus subtilis* but Ensure Its Efficient Utilization

**DOI:** 10.1371/journal.pgen.1002561

**Published:** 2012-03-22

**Authors:** Christopher D. A. Rodrigues, Elizabeth J. Harry

**Affiliations:** The ithree institute, School of Medical and Molecular Biosciences, University of Technology, Sydney, Australia; Agency for Science, Technology, and Research, Singapore

## Abstract

Precise temporal and spatial control of cell division is essential for progeny survival. The current general view is that precise positioning of the division site at midcell in rod-shaped bacteria is a result of the combined action of the Min system and nucleoid (chromosome) occlusion. Both systems prevent assembly of the cytokinetic Z ring at inappropriate places in the cell, restricting Z rings to the correct site at midcell. Here we show that in the bacterium *Bacillus subtilis* Z rings are positioned precisely at midcell in the complete absence of both these systems, revealing the existence of a mechanism independent of Min and nucleoid occlusion that identifies midcell in this organism. We further show that Z ring assembly at midcell is delayed in the absence of Min and Noc proteins, while at the same time FtsZ accumulates at other potential division sites. This suggests that a major role for Min and Noc is to ensure efficient utilization of the midcell division site by preventing Z ring assembly at potential division sites, including the cell poles. Our data lead us to propose a model in which spatial regulation of division in *B. subtilis* involves identification of the division site at midcell that requires Min and nucleoid occlusion to ensure efficient Z ring assembly there and only there, at the right time in the cell cycle.

## Author Summary

How organisms regulate biological processes so that they occur at the correct place within the cell is a fundamental question in research. Spatial regulation of cell division is vital to ensure equal partitioning of DNA into newborn cells. Correct positioning of the division site at the cell centre in rod-shaped bacteria is generally believed to occur via the combined action of two factors: (i) nucleoid (chromosome) occlusion and (ii) a set of proteins known collectively as the Min system. The earliest stage in bacterial cell division is the assembly of a ring, called the Z ring, at the division site. Nucleoid occlusion and Min work by preventing Z ring assembly at all sites along the cell other than the cell centre. Here we make the surprising discovery that, in the absence of both these factors, Z rings are positioned correctly at the division site, but there is a delay in this process and it is less efficient. We propose that a separate mechanism identifies the division site at midcell in rod-shaped bacteria, and nucleoid occlusion and Min ensure that the Z ring forms there and only there, at the right time and every time.

## Introduction

Mechanisms that regulate cell division in time and space are of fundamental importance to biology because they ensure equal partitioning of DNA into newborn cells. Failure to do so can lead to cell death. The earliest observable event in cell division in rod-shaped bacteria such as *Escherichia coli* and *Bacillus subtilis* is the polymerization of the highly conserved tubulin-like protein, FtsZ, to form a contractile structure called the Z ring, at midcell [1]–[6]. The Z ring then recruits several division proteins to form the division complex, known as the divisome, to enable cytokinesis. In this way FtsZ acts as a so-called founder protein that recognizes a sub-cellular location, and instructs other proteins to assemble there through a series of protein interactions [7]. The key question concerning the regulation of cell division is therefore, what dictates the sub-cellular recruitment of this founder protein, FtsZ, precisely to midcell?

For many years the paradigm for division site positioning in rod-shaped bacteria such as *E. coli* and *B. subtilis* has been that it is determined through the combined action of the Min system and nucleoid occlusion. Both systems negatively regulate Z ring formation by preventing Z rings forming anywhere in the cell except midcell. The Min system prevents Z rings assembling at the poles where there is little or no DNA, whereas nucleoid occlusion prevents Z rings assembling over the nucleoid or chromosome [1]–[3], [8]. It is generally believed that when chromosomes segregate, the DNA-free space created at midcell relieves nucleoid occlusion, allowing a Z ring to form precisely at this site [9]–[14].

In *E. coli*, the Min system consists of three different proteins: MinC, MinD and MinE [15], [16]. The *B. subtilis* Min system comprises four proteins: MinC, MinD, MinJ and DivIVA, with MinC and MinD being homologs of the corresponding *E. coli* counterparts [17]–[21]. In both organisms, MinC is the primary inhibitor of the system; and it appears to inhibit Z ring formation by interacting and destabilizing FtsZ polymers directly [16], [22], [23]. Recent evidence suggests that MinC inhibits FtsZ polymer assembly by preventing lateral interactions between FtsZ protofilaments in *B. subtilis* and *E. coli* [24], [25]. Spatial regulation of Z ring assembly by the MinC protein in both organisms relies on its dynamic localization throughout the cell cycle [26]–[28].

Nucleoid occlusion in *B. subtilis* and *E. coli* involves the Noc and SlmA proteins, respectively. These proteins appear to perform similar roles, but are not similar in protein sequence [29]–[31]. Both of them bind to DNA and inhibit Z ring assembly over the chromosome [29], [30], [32]–[34]. Noc and SlmA bind to specific DNA sequences that are particularly sparse or absent near the terminus region of the chromosome [32]–[34]. It is proposed that as the round of replication nears completion and the terminus region occupies a midcell location, Noc and SlmA move away from midcell as the bulk of the chromosomes segregate, allowing a Z ring to form there [32]–[34]. The critical role of both of these proteins appears to be in preventing guillotining of the DNA by the septum when chromosome replication or segregation is perturbed [29], [30].

Interestingly, neither the Min system nor the Noc/SlmA proteins are essential in *E. coli* or *B. subtilis*. Furthermore, it has been shown previously that MinC and MinD are not required for the precise positioning of Z rings at midcell in *B. subtilis* [35]. However in *B. subtilis* and *E. coli* cells deprived of both Noc or SlmA proteins and the Min system, Z rings form very infrequently and division is defective, resulting in long cells [29], [30]. It has been proposed that this is due to the ability of FtsZ to form polymers at multiple locations along the length of the cell, including over nucleoids, none of which can accumulate enough FtsZ to form a ring [29], [30]. Thus the prevailing belief is that Noc/SlmA and the Min proteins together are primarily responsible for identifying the division site in bacteria by allowing Z rings to form precisely at midcell [1]–[3], [8]. However, while it is clear that these proteins play an important role in influencing placement of the Z ring at the correct site, whether they are solely responsible for the precise positioning of the Z ring at midcell remains to be tested.

Cumulative evidence suggests that mechanisms in addition to Noc/SlmA and the Min system are involved in positioning the Z ring at midcell. Firstly, in Noc^−^ Min^−^ *B. subtilis* and SlmA^−^ Min^−^ *E. coli* double-mutant cells overproducing FtsZ, division is partially restored and Z ring positioning appears biased towards inter-nucleoid spaces [29], [30]. The significance of this bias however has never been investigated and thus it is still not known to what extent the double-mutant defect actually affects Z ring positioning. Given that neither *noc* or *minCD* single mutants of *B. subtilis* affect Z ring positioning at midcell, it brings into question the exact degree to which the combined action of Noc and the Min system contribute to division site positioning. Indeed, previous studies point to a Noc-independent link between the early stages of DNA replication and division site positioning in *B. subtilis* [36]–[38]. In addition many bacteria do not have the inhibitory Noc (or SlmA) or Min homologues [1], [8]. The observations that Noc is not essential in *Staphylococcus aureus*, which does not contain a Min system [39]; and that two proteins in *Streptomyces coelicolor*, SsgA and SsgB, positively regulate FtsZ positioning between nucleoids during sporulation [40], highlights the distinct possibility that in other bacteria, including *B. subtilis*, other mechanisms are involved in establishing the position of the division site at midcell.

Here we make use of the outgrown spore system to investigate the extent to which Min and nucleoid occlusion are responsible for positioning the Z ring in *B. subtilis*. We show that, remarkably, in the absence of both Min and Noc, Z rings are positioned precisely at midcell; although their assembly is delayed and less efficient. We also show that in the absence of both Min and any type of nucleoid occlusion, there is a substantial preference for Z rings to form precisely at midcell between two nucleoids rather than between the nucleoid and the pole. Our data strongly support a model in which correct positioning of the Z ring at the division site in *B. subtilis* is not solely determined by the combined effect of Min and nucleoid occlusion. Furthermore the data raises the possibility that the primary role of Min and nucleoid occlusion is to ensure the efficient utilization of the division site at midcell in *B. subtilis* by ensuring Z ring placement there, while other mechanisms are responsible for the actual identification of this site.

## Results

### Z rings are positioned precisely at midcell in the absence of both *noc* and *minCD*

In the absence of Noc and the Min system, Z rings do form in vegetatively-growing cells, albeit very infrequently [29]. However it remains unclear whether, in these long cells, Z rings form precisely at the division site under these conditions. The outgrown spore system of *B. subtilis* allows an accurate measurement of Z ring position during the first one or two cell cycles following germination. We therefore examined the precision of Z ring positioning in the absence of MinCD and Noc in outgrown *B. subtilis* spores.

We created a *minCD noc* double-mutant strain (SU681), by introducing a *minCD::cat* cassette into a strain containing a *noc::tet* cassette (SU656; for all strains refer to Table 1). At 30°C, vegetatively growing cells of the *minCD noc* double-mutant were on average three-fold longer (11.8±0.54 µm, mean cell length ± SEM, Figure S1A and S1E) than wild-type cells grown under the same conditions (3.8±0.06 µm; Figure S1A and S1C). At 37°C, the double-mutant cells were even longer as previously reported [29] but in similar proportion relative to wild-type cells at the same temperature (4.7±0.08 µm and 18.1±0.9 µm, respectively; Figure S1B, S1D and S1F). Similar cell lengths were obtained when MinD was depleted in a Noc^−^ background (data not shown). Spores of the *minCD noc* double-mutant strain (SU681), from now on referred to as the double-mutant strain, were obtained despite the lower sporulation efficiency compared to the wild-type strain, SU5 (data not shown).

**Table 1:** *B. subtilis* strains.

Strain (construction: donor→recipient)	Genotypea)	Reference or source
**1284**	*trpC2, minD::pSG1737-minCD'-lacZ P_spac_-minCD (ermC), noc::tet*	Wu and Errington (2004)
**SU5**	*trpC2*	E. Nester
**SU46**	*trpC2, thyA, thyB, dna-1*	N.Sueoka
**SU456**	*trpC, ftsZ:: P_spac_-ftsZ (phleo)*	Jensen et al, (2005)
**SU492**	*trpC2, amyE::P_xyl_-ftsZ-yfp (spec)*	Lab stock
**SU558**	*trpC2, amyE::P_spachy_-ftsZ (neo)*	Lab stock
**SU561**	*trpC2, minCD::cat*	Lab stock
**SU656**	*trpC2, noc::tet*	Lab stock
**SU657**	*trpC2, noc::tet, amyE::P_xyl_-ftsZ-yfp (spec)*	Lab stock
**SU661 (SU46→SU5)** b)	*trpC2^+^, dna-1*	This work
**SU662 (SU456→SU661)**	*trpC2^+^, dna-1, ftsZ::P_spac_-ftsZ (phleo)*	This work
**SU663 (SU561→SU657)**	*trpC2, amyE:: P_xyl_-ftsZ-yfp (spec), noc::tet, minCD::cat*	This work
**SU671 (SU492→SU662)**	*trpC2^+^, dna-1, ftsZ::P_spac_-ftsZ (phleo), amyE::P_xyl_-ftsZ-yfp (spec)*	This work
**SU678 (SU561→SU671)**	*trpC2^+^, dna-1, ftsZ::P_spac_-ftsZ (phelo), amyE::P_xyl_-ftsZ-yfp (spec), minCD::cat*	This work
**SU680 (SU656→SU678)**	*trpC2^+^, dna-1, ftsZ::P_spac_-ftsZ (phleo), amyE::P_xyl_-ftsZ-yfp (spec) minCD::cat*, *noc::tet*	This work
**SU681 (SU561→SU656)**	*trpC2, noc::tet, minCD::cat*	This work
**SU683 (SU656→SU671)**	*trpC2^+^, dna-1, ftsZ::P_spac_-ftsZ (phleo), amyE::P_xyl_-ftsZ-yfp (spec), noc::tet*	This work
**SU684 (SU561→SU558)**	*trpC2, amyE::P_spachy_-ftsZ (neo), minCD::cat*	This work
**SU685 (SU656→SU684)**	*trpC2, amyE::P_spachy_-ftsZ (neo), minCD::cat, noc::tet*	This work

a) Antibiotic resistance genes are expressed as follows: *cat*, chloramphenicol; *spc*, spectinomycin, *tet*, tetracycline; *neo*, neomycin; *ermC*, erythromycin; *phleo*, phleomycin.

b) This strain was obtained by congression and contains a reversion of the *trpC2* marker - *trpC2^+^*.

**Figure 1:**
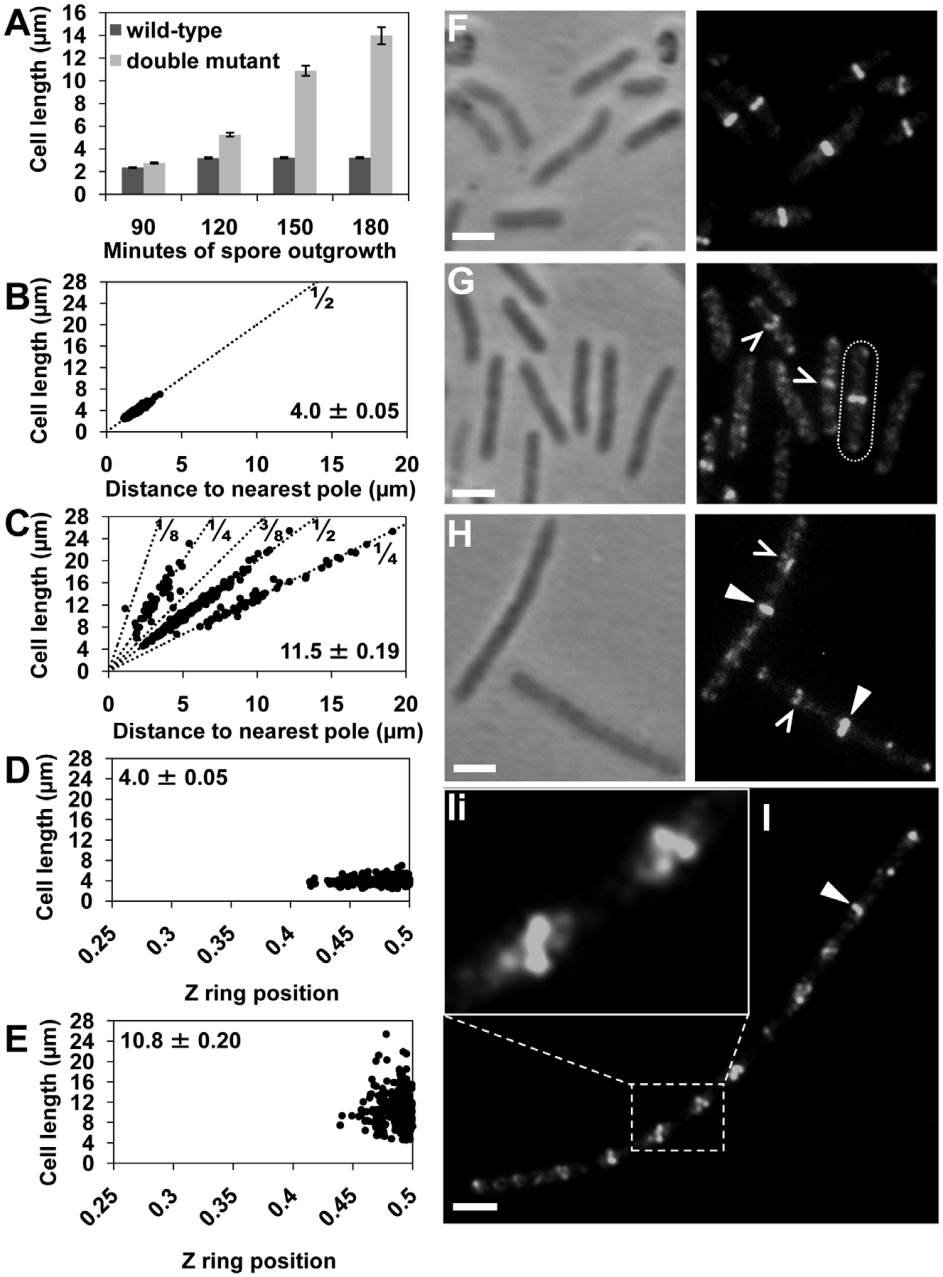
Z rings form precisely at midcell in the *noc minCD* double mutant. Spores of the double-mutant (SU681) and wild-type (SU5) strain were germinated in PAB at 34°C. Cells were fixed in ethanol or prepared for immunofluorescence to visualize FtsZ. (A) Mean length of the wild-type and double-mutant outgrown cells collected at 90, 120, 150 and 180 min after spore germination and prepared for ethanol-fixation. Error bars are µm ± SEM. (B and C) Z ring position plotted in relation to the predicted position of potential division sites (dotted lines; 1/2, 1/4, 1/8, 3/8) in the wild-type (B) and double mutant (C) strains at 150 min of spore outgrowth. Bottom-left corner shows the mean cell length of the cells plotted (µm ± SEM). (D and E) Z ring position at the first (medial) division site in the wild-type (D) and the double-mutant (E) cells at 120 min and 150 min of spore outgrowth, respectively. Top left-hand corner shows the average cell length of the cells plotted (µm ± SEM). (F and G) FtsZ localization in wild-type (F) and double-mutant (G) at 120 min of spore outgrowth. Cell containing a midcell Z ring in the double-mutant strains is outlined. (H and I) FtsZ localization in the double-mutant at 150 min (H) and at 180 min (I) of spore outgrowth. (Ii) Amplification of the white rectangle in (I) showing helical-like patterns of FtsZ. Carets point to accumulations of FtsZ at division sites and triangles point to Z rings in the double mutant. Images are phase contrast (left), and FtsZ immunofluorescence (right). Scale bars are 2 µm.

Using immunofluorescence microscopy we examined Z ring positioning in outgrown spores of the double-mutant strain at 34°C (the optimum temperature for spore germination), and compared it to wild-type cells (SU5) outgrown under the same conditions. If Min and Noc are solely responsible for determining the division site in this organism, then we would expect that no or very few cells will form Z rings precisely at midcell. At 120 min after germination, wild-type cells had an average cell length of 3.20±0.06 µm (Figure 1A). The vast majority of these cells (81%) contained a Z ring exclusively at midcell, with 91% of these positioned between 0.45 and 0.5 (Figure 1D and 1F). Z ring position is defined by the distance from the Z ring to nearest pole, divided by the cell length, with 0.5 being exactly midcell. At the same time point, the double-mutant strain (SU681) had an average cell length of 5.3±0.12 µm (Figure 1A) and the frequency of outgrown cells with Z rings was extremely low (3.5%; 12 out of 340 cells counted). Most remarkably however, 8 of these cells had a bright Z ring located precisely at midcell (see delineated cell in Figure 1G), with the other 4 cells having polar Z rings. Furthermore, several other cells had a distinct accumulation of FtsZ in their central region (see carets in Figure 1G).

At 150 min of spore outgrowth, cells of the double-mutant strain reached an average length of 10.9±0.45 µm (Figure 1A) and the proportion of cells with Z rings increased significantly to 30% allowing us to measure Z ring positioning in a larger number of cells. Remarkably, at this time point, of the cells containing a Z ring, 60% had one exclusively at midcell (see triangles in Figure 1H). The remaining 40% had Z rings that were positioned either at the poles (5%), at positions other than the pole or midcell (27%) or had more than one Z ring (8%). The remainder of the cell population contained diffuse patterns of FtsZ that appeared as faint bands, helical-like structures, or accumulations of FtsZ as previously reported [29]. We measured the precise position of the medially-located Z rings in the double-mutant strain relative to wild-type Z rings at 120 min. We used 120 min for wild-type cells (SU5) for this comparison as this is when we first observe Z rings after germination. In the double-mutant strain, essentially all these Z rings were located between 0.45–0.5 (99%; Figure 1E; compare with Figure 1D). In other words, Z rings that assemble at midcell in the absence of both Min and Noc are at least as precisely positioned as those observed in wild-type cells.

To determine the position of all the non-polar Z rings in the absence of both Noc and Min at 150 min, we plotted their position on a graph showing the theoretical division sites along the length of the bacillus rod; with 1/4 representing the second division sites and 1/8 and 3/8 representing the third division sites as described previously ([41]; Figure 1C). As expected, in wild-type (SU5) cells all Z rings were positioned at the 1/2 position (Figure 1B). Interestingly, in the double mutant the non-polar Z rings were either positioned at midcell or at the second division sites (Figure 1C). In other words, in the absence of both Min and Noc, there is a preference for Z rings to form at the first division site at midcell, as well as at the second division site.

At 180 min the average cell length of the double-mutant increased to 13.9±0.74 µm (Figure 1A), and the majority (70%) of cells contained at least one Z ring (Figure 1I; see white triangle). These cells resemble the vegetative phenotype of the *minCD noc* double-mutant. Importantly, septation was often observed along these longer cells, showing that at least some of these Z rings are functional (data not shown). Moreover, these longer cells often contained a number of accumulations of FtsZ regularly spaced along their length (Figure 1I). These non-ring localizations of FtsZ are likely precursors to the Z rings at the division site [42], [43].

In summary, the above data establishes that in the absence of both Noc and Min, in addition to cells forming polar Z rings as expected, a significant number of Z rings can form at midcell as well as at future division sites. Furthermore, the precision with which the Z ring is positioned at midcell is unchanged in the absence of both Noc and Min. So, while these factors play an important role in determining where a Z ring will form in the cells, other mechanisms must exist to ensure precise placement of the Z ring at midcell in *B. subtilis*.

### The absence of both *noc* and *minCD* results in a severe delay in midcell Z ring assembly

While Z rings assembled precisely at midcell in the absence of both Noc and the Min system, their assembly was significantly delayed and resulted in much fewer Z rings at midcell compared to wild-type cells at specific times following spore germination. This delay was accompanied by the ability of FtsZ to accumulate at the second division site. To determine whether Z rings could form at midcell earlier in the cell cycle in the double-mutant strain, and maintain their precision in the absence of both Min and Noc, we increased the cellular level of wild-type FtsZ in the double-mutant during spore outgrowth. This was achieved by introducing a second copy of *ftsZ* as an IPTG-inducible *P_spachy_-ftsZ* construct, integrated at the *amyE* locus of the chromosome of the double-mutant to give SU685, and in wild-type cells to give SU558. In a wild-type background, high cellular levels of FtsZ can inhibit division [44]. We therefore performed these experiments using the highest concentration of IPTG (0.05 mM) that allowed essentially normal division of SU558 cells during vegetative growth (see Figure S2), and spore outgrowth in the wild-type background. FtsZ overproduction with 0.05 mM IPTG significantly rescued the division phenotype of vegetatively-growing double-mutant (SU685) cells both at 30°C and 37°C (see Figure S2 and Figure S3). Western blot analysis confirmed that FtsZ was overproduced 1.3-fold in both the double-mutant and wild-type strains containing *P_spachy_-ftsZ* in the presence of 0.05 mM IPTG compared to the equivalent strains that did not contain P*_spachy_-ftsZ* (Figure S2E).

**Figure 2:**
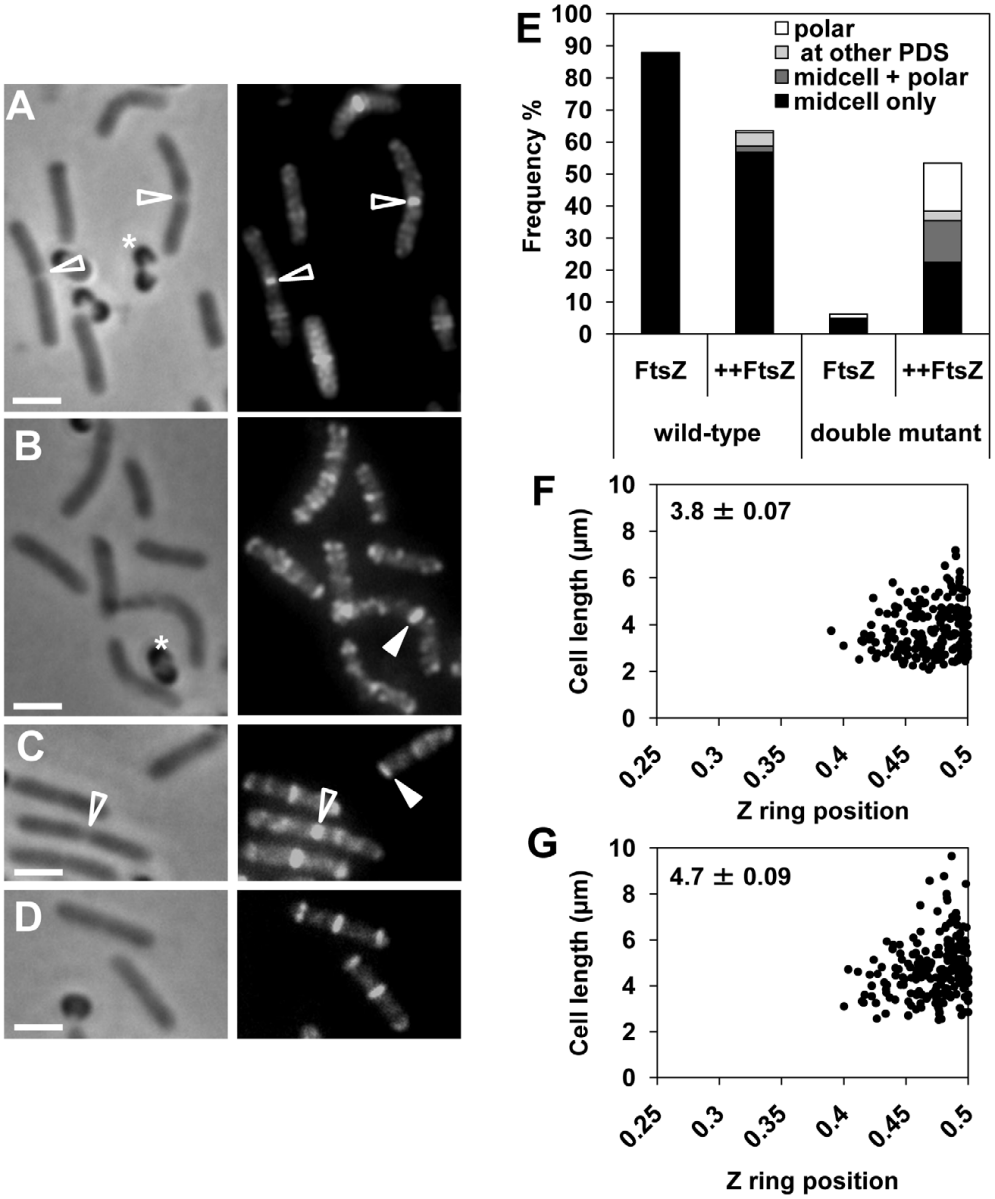
FtsZ overproduction reduces the delay in Z ring assembly at midcell in the *noc minCD* double mutant. Spores of double-mutant (SU685) and the wild-type (SU558) strains, containing P*_spachy_-ftsZ* integrated at *amyE*, were outgrown at 34°C in PAB with 0.05 mM IPTG or without IPTG and collected at 120 min for visualization of FtsZ using immunofluorescence microscopy. (A to D) FtsZ localization in wild-type cells germinated with IPTG at 120 min (A), in the double-mutant without IPTG at 120 min (B), and in the double-mutant with IPTG at 120 min (C and D). Images are phase-contrast (left), and FtsZ immunofluorescence (right). Open triangles point to constricting Z rings and closed triangles point to examples of Z rings. Stars denote spore coats. Scale bars are 2 µm. (E) Frequency of outgrown spores that contain Z rings for the wild-type and double-mutant strains. Z ring frequencies are shown in the presence (++FtsZ) or absence (FtsZ) of IPTG (n>400). The height of the bar shows the total frequency of cells with Z rings. Each bar is divided into percentages of cells with a single midcell Z ring (midcell only, black bar), cells with more than one Z ring (midcell+polar, dark grey bar), a single Z ring located at a future (potential) division site (PDS, light grey bar) and a single polar Z ring (polar, white bar). Average cell lengths of the whole population in the wild-type background were 2.8±0.05 (µm ± SEM) with IPTG (++FtsZ) and 2.6±0.06 without IPTG (FtsZ); and in the double-mutant background were 3.1±0.07 with IPTG (++FtsZ) and 3.3±0.07 without IPTG (FtsZ). (F and G) Z ring positioning at the first (medial) division site in the wild-type (F) and double-mutant (G) outgrown spores overproducing FtsZ. Top left-hand corner denotes the average cell length of the cells plotted (µm ± SEM).

FtsZ overproduction in the wild-type background (SU558) during spore outgrowth resulted in 64% of the cells in the population containing Z rings at 120 min. This is lower than when FtsZ is not overproduced in wild type cells (88%; Figure 2E). The reason for this decrease in Z ring frequency is not known, however it could be due to the observed assembly of midcell Z rings slightly earlier in the cell cycle (at 90 min rather than 120 min) under these conditions, leading to early division and subsequently an increase in the number of newborn cells in the population at 120 min that do not yet have a Z ring (data not shown). Importantly, of the wild-type FtsZ-overproducing cells containing Z rings (64%), the vast majority (89%) contained a Z ring exclusively at midcell (Figure 2A and 2E), with a small number of cells containing polar Z rings (1.6%). Cells with Z rings located at the quarter positions, and cells with more than one Z ring represented 9.4% of the total population of cells with Z rings (Figure 2E). In the double-mutant even at 120 min following spore germination FtsZ overproduction resulted in a dramatic increase in the frequency of cells containing a Z ring; from 6% with no overproduction, to 53% in the presence of IPTG (Figure 2E; compare Figure 2B to 2C and 2D). Of the cells containing Z rings (318 cells counted), 42% had a Z ring exclusively at midcell (Figure 2C and 2E), 28% a single polar Z ring (Figure 2E and 2C, see closed triangle), and 6% contained a Z ring at the quarter positions (Figure 2E; PDS). The remainder of these cells (24%) contained more than one Z ring - predominantly a Z ring at midcell and another Z ring at the pole (Figure 2D and 2E). Moreover, the precision of Z ring positioning at midcell in these FtsZ-overproducing mutant cells was the same as for wild type cells (only cells with a single medially-located Z rings were measured; compare Figure 2F and 2G). These results demonstrate that overproduction of FtsZ in the absence of both Min and Noc significantly reduces the delay in midcell Z ring assembly, and maintains the precision of Z ring placement at this site.

In the above experiments Z rings were visualized using immunofluorescence, which does not preserve the nucleoid well. To confirm that Z rings actually formed between nucleoids in the double mutant when FtsZ is overproduced, we co-visualized the Z ring and the nucleoid in live cells of both the double-mutant and wild-type strains. We constructed both wild-type (SU492) and double-mutant strains (SU663) that contain a xylose-inducible copy of *ftsZ-yfp* located at *amyE*, allowing visualization of the Z ring in live cells in the presence of xylose. Western blot analysis confirmed that in the presence of 0.5% xylose, total cellular FtsZ levels (FtsZ-YFP plus native FtsZ) were overproduced to approximately 1.25-fold in the double-mutant (SU663) and wild-type (SU492) vegetatively-growing cells, relative to FtsZ levels in the equivalent strains not containing P*_xyl_-ftsZ-yfp* (Figure S4D). As expected, during vegetative growth, while the addition of 0.5% xylose did not cause any significant changes to the wild-type cell length, it did result in a significant rescue of cell division in the double-mutant (Figure S4A).

**Figure 3:**
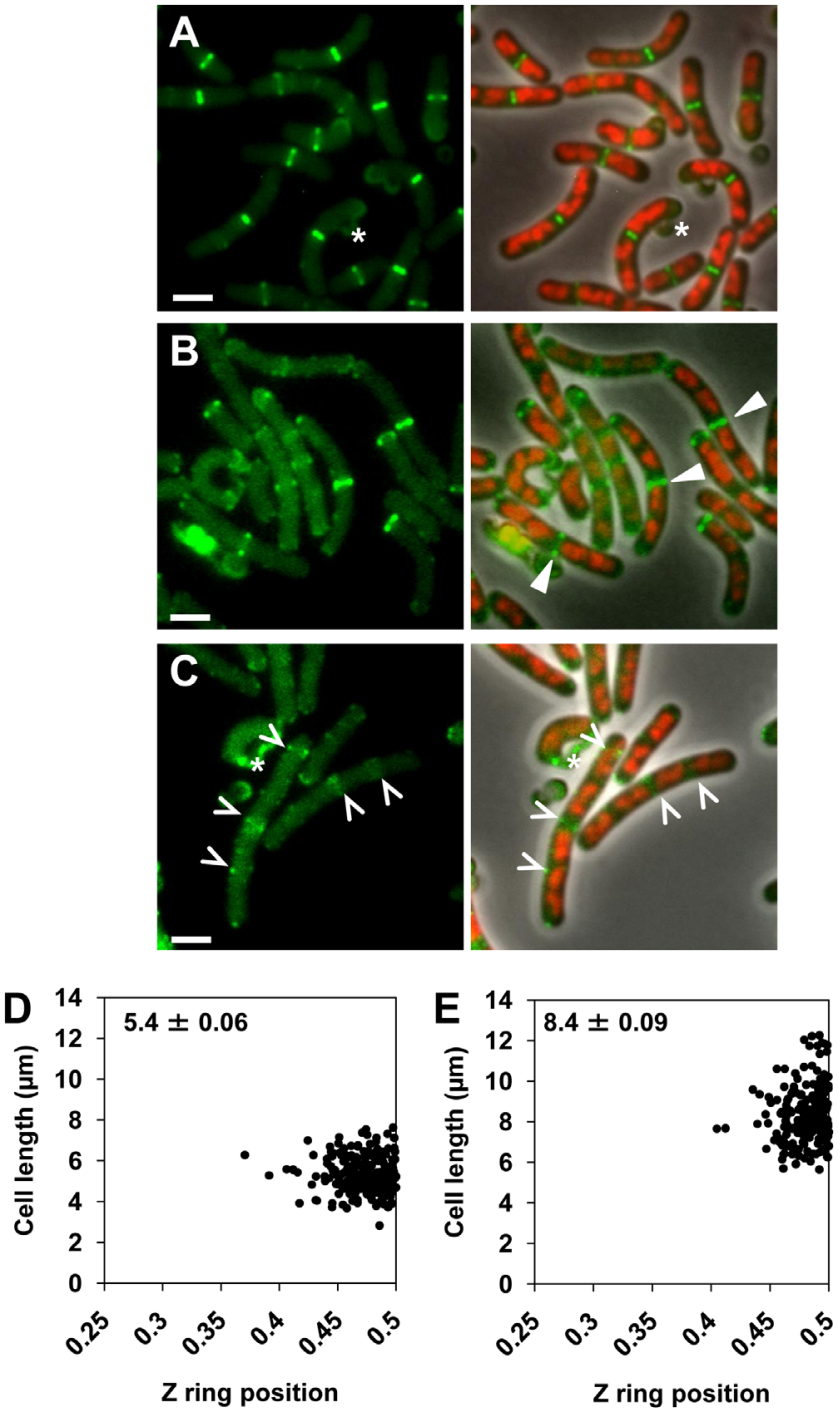
Midcell Z rings form precisely between replicating nucleoids in the *noc minCD* double mutant. Spores of wild-type (SU492) and double-mutant (SU663) strains, both containing P*_xyl_-ftsZ-yfp* integrated at the *amyE* locus, were outgrown at 34°C in PAB with 0.5% xylose and collected for live cell analysis at 120 min of outgrowth. (A and B) Z ring localization in wild-type (A) and double-mutant (B) cells. (C) Accumulations of FtsZ present in the double-mutant cells. Images are FtsZ-YFP pseudo-coloured in green (left) and phase contrast overlay (right) of pseudo-coloured DAPI (red) and pseudo-coloured FtsZ-YFP (green). Carets point to accumulations of FtsZ and white triangles point to Z rings between nucleoids. Stars denote fluorescent spore coats. Scale bars are 2 µm. (D and E) Z ring positioning at the first (medial) division site in the wild-type background (D) and double-mutant (E) outgrown spores, both overproducing FtsZ-YFP. Top left-hand corner denotes the average cell length of cells plotted (µm ± SEM).

Examination of double-mutant and wild-type cells overproducing FtsZ (as FtsZ and FtsZ-YFP) confirmed that all Z rings do indeed form between replicating nucleoids during spore outgrowth (Figure 3A and 3B; see triangles) and vegetative growth (Figure S4B and S4C). Moreover, non-ring localizations of FtsZ, that are likely precursors to Z rings at the division site [42], [43], were also readily observed between nucleoids in live cells of the double mutant (see carets, Figure 3C). As with overproduction of native FtsZ, overproduction of FtsZ using FtsZ-YFP rescued the timing of Z ring assembly, although to a slightly less extent than just FtsZ (data not shown). At 120 min, in the double mutant strain overproducing FtsZ-YFP (SU663), 34% of the outgrown spores had Z rings, 60% of which had a Z ring exclusively at the medial division site, 30% had a single polar Z ring and the remaining 10% had more than one Z ring. An analysis of Z ring positioning in live outgrown spores germinated in the presence of 0.5% xylose at 120 min of outgrowth confirmed that in double-mutant cells that had a Z ring exclusively at midcell, it was positioned with wild-type precision (Figure 3D and 3E).

In summary, these results show that overproduction of FtsZ in the complete absence of Noc and the Min system, can significantly rescue the observed delay in Z ring assembly at midcell in these cells, enabling Z rings to form at midcell earlier in the cell cycle and division to occur at midcell more efficiently. This suggests that while Min and Noc are not required for the precise positioning of a Z ring at midcell, they are needed for the efficient assembly of Z rings at this site, as well as preventing Z rings forming at other division sites.

### The division site is positioned at midcell independently of the Min system and all nucleoid occlusion

Our data show that in the absence of Noc and the Min system, midcell Z rings still form precisely at midcell. We considered the possibility that, in the absence of Noc and Min, another nucleoid occlusion factor ensures precise positioning of the Z ring at midcell [29], [45]. To test this, we developed an approach that allowed replicated nucleoids to separate substantially after a single round of chromosome replication, such that there is no DNA (no nucleoid occlusion) in the central region of the cell. We then induced FtsZ production to determine whether a Z ring would assemble there. We performed these experiments in the absence of the Min system, the Noc protein, or both. Assembly of Z rings precisely at midcell under these conditions would support the idea of a nucleoid occlusion-independent mechanism for Z ring positioning. Furthermore, preferential positioning of the Z ring precisely at midcell as opposed to the cell poles under these conditions would argue strongly for the identification of a specific site at midcell for Z ring assembly in *B. subtilis* that does not require Min or nucleoid occlusion.

**Figure 4:**
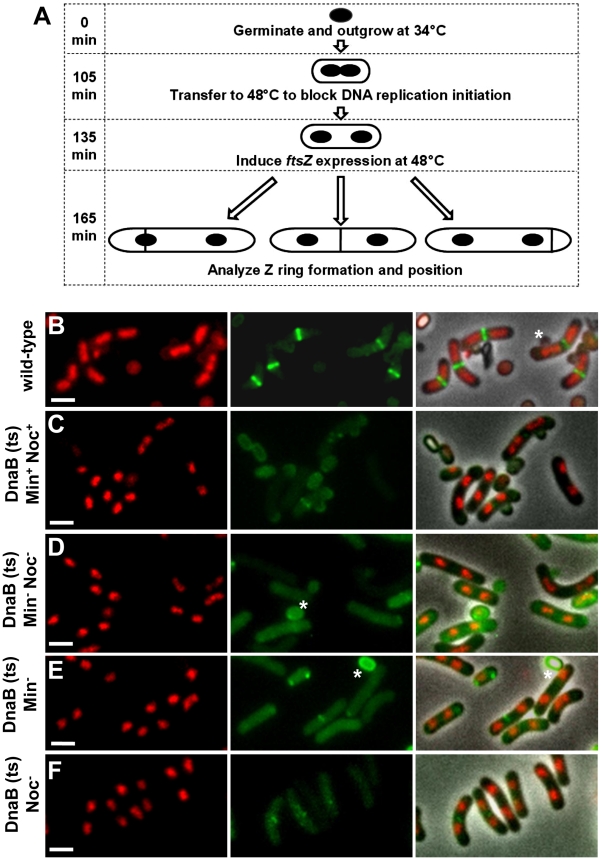
Experimental approach and separation of nucleoids after one round of replication, prior to production of FtsZ. (A) Diagram of experimental approach (see Materials and Methods for complete details). Spores of the *dnaB* (ts) strains containing P*_spac_-ftsZ* and P*_xyl_-ftsZ-yfp* [strains SU671 (Min^+^, Noc^+^), SU678 (Min^−^), SU680 (Min^−^, Noc^−^), and SU683 (Noc^−^)] were germinated in GMD at 34°C and then transferred to 48°C to prevent re-initiation of DNA replication and allow separation of replicated nucleoids. IPTG (1 mM) and xylose (0.01%) were then added. Cells were removed and prepared for live visualization of Z rings and nucleoids. (B) Representative images of Z rings and nucleoids in the wild-type control strain SU492 (DnaB^+^). Spores of this strain were germinated in GMD with 0.01% xylose at 34°C for 105 min, transferred to 48°C for 30 min and then collected for analysis. These conditions differ from that of the test strains to allow visualization of Z rings in the first round of replication. (C to F) Representative images of cells of the *dnaB* (ts) strains containing two separated nucleoids, collected prior to induction of *ftsZ* expression with IPTG, when both MinCD and Noc are present (C), when both MinCD and Noc are absent (D), when MinCD is absent (E), when Noc is absent (F). Images are DAPI (nucleoid) pseudo-coloured in red (left), FtsZ-YFP pseudo-coloured in green (middle) and phase-contrast fluorescence overlay (right). Stars denote autofluorescent spore coats. Scale bars are 2 µm.

Our experimental approach is shown in Figure 4A (refer to legend). We used the temperature sensitive *dnaB* mutant, *dna-1*, to allow only one round of DNA replication during spore outgrowth. This mutation provides a complete block to initiation of DNA replication at the non-permissive temperature (48°C; [46]). Spores were germinated at the permissive temperature for long enough to allow initiation of one round of replication, and then shifted to the non-permissive temperature to prevent initiation of a second round of replication. This allows two replicated nucleoids to separate substantially prior to induction of FtsZ production. We also used an alternative approach following a similar rationale. This involved spore outgrowth at 34°C, but instead of inhibiting DNA replication using the temperature-sensitive DNA replication mutant *dna-1*, we added HPUra, a potent inhibitor of *B. subtilis* DNA polymerase III [47], at pre-determined time points to create a significant population of cells with two separated chromosomes, prior to inducing FtsZ production. This alternative approach was performed in RecA^−^ cells to avoid the SOS response-mediated inhibition of Z ring formation and division [48]–[50]. The results we obtained were essentially the same as those obtained using the *dna-1* approach described below (data not shown).

**Figure 5:**
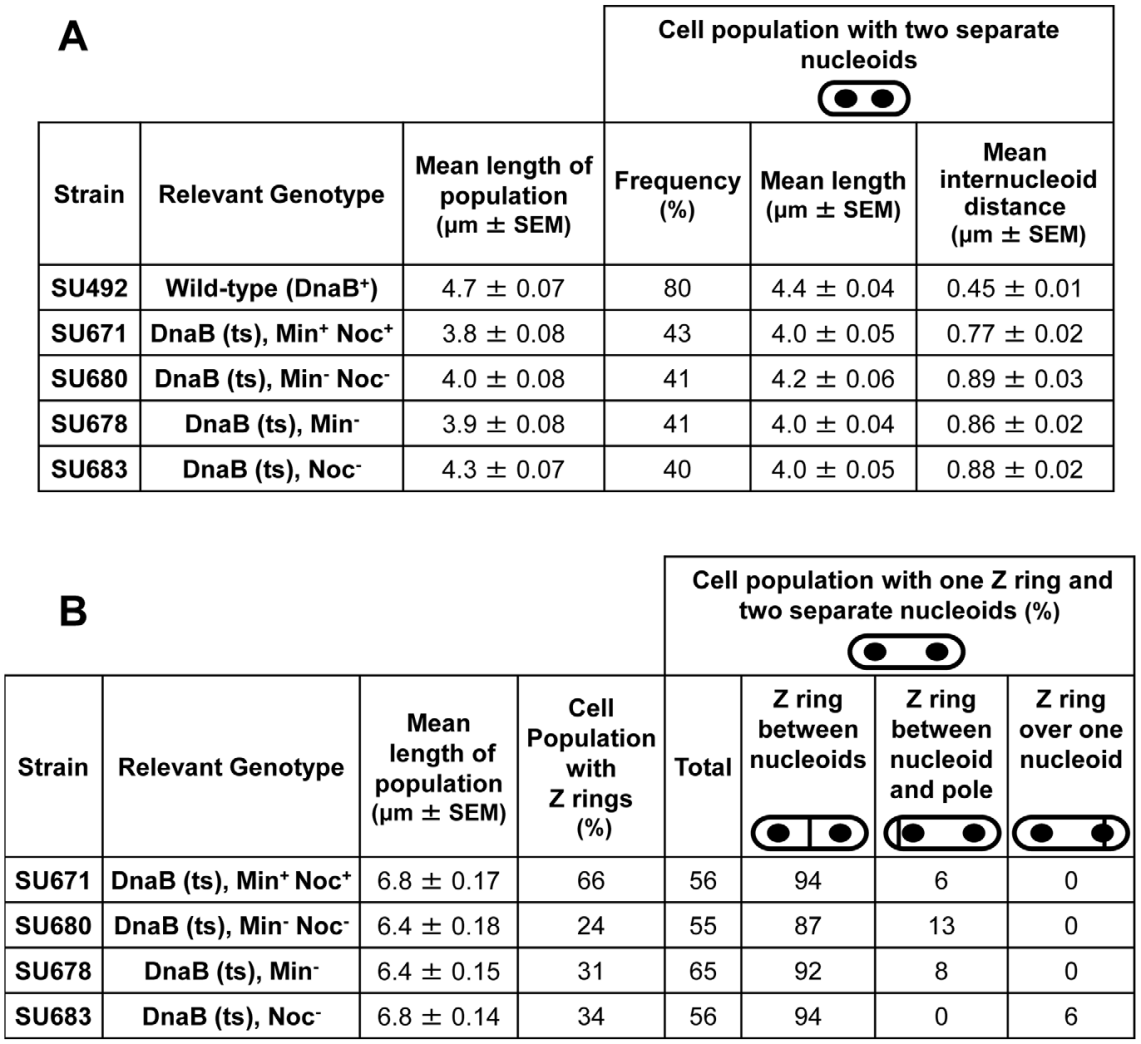
Separation of nucleoids and Z ring positioning in outgrown spores of *dnaB* (ts) strains. Refer to Figure 4A for experimental design. (A) Separation of nucleoids in cells collected at 135 min after the start of germination, prior to induction of *ftsZ* expression. Columns read left to right; e.g. DnaB (ts), Min^+^Noc^+^: the mean cell length of the population was 3.8 µm; 43% of the population contained two separated nuceloids; cells with two nucleoids had a mean length of 4.0 µm; the mean internucleoid distance in cells with two nucleoids was 0.77 µm. (B) Z ring assembly and positioning in cells collected at 165 min after the start of germination, 30 min after induction of *ftsZ* expression. Columns read left to right: e.g. DnaB (ts), Min^+^Noc^+^: the mean cell length of the population was 6.8 µm; Z rings were present in 66% of cells, out of which 56% had one Z ring and two nucleoids. Of these cells with two nucleoids, 94% had a Z ring between two nucleoids and 6% had a Z ring between the nucleoid and the pole. SEM is standard error of the mean.

Using the *dna-1* approach shown in Figure 4A, for all strains containing the *dnaB* (ts) mutation, at least 40% of cells contained two replicated nucleoids that had separated to produce a clear DNA-free gap in the central region of the cell at 135 min, prior to induction of *ftsZ* expression (Figure 5A and Figure 4C–4F). The remaining cell populations contained either a single nucleoid (no replication) or more than two nucleoids (more than one initiation; data not shown). *ftsZ* expression was then induced from P*_spac_-ftsZ* (controlling *ftsZ* expression at the native locus) with 1 mM IPTG to allow Z ring assembly. These cells also contained P*_xyl_-ftsZ-yfp* (located at *amyE*) induced minimally (with 0.01% xylose) to enable visualization of the Z ring in live cells. To ensure that nucleoid occlusion was completely relieved at midcell before Z ring assembly had begun, we induced *ftsZ* expression when the DNA-free gap in cells with two nucleoids was on average almost twice as large as it is in wild-type outgrown cells (SU492, DnaB^+^) that were grown out for 105 min at 34°C then shifted to 48°C for 30 min (0.45 µm, see Figure 4B; Figure 5A; compare the DNA-free space in Figure 4B to Figure 4C–4F). As expected, in the absence of xylose and IPTG we very rarely observed Z rings (using immunofluorescence; Figure S5).

**Figure 6:**
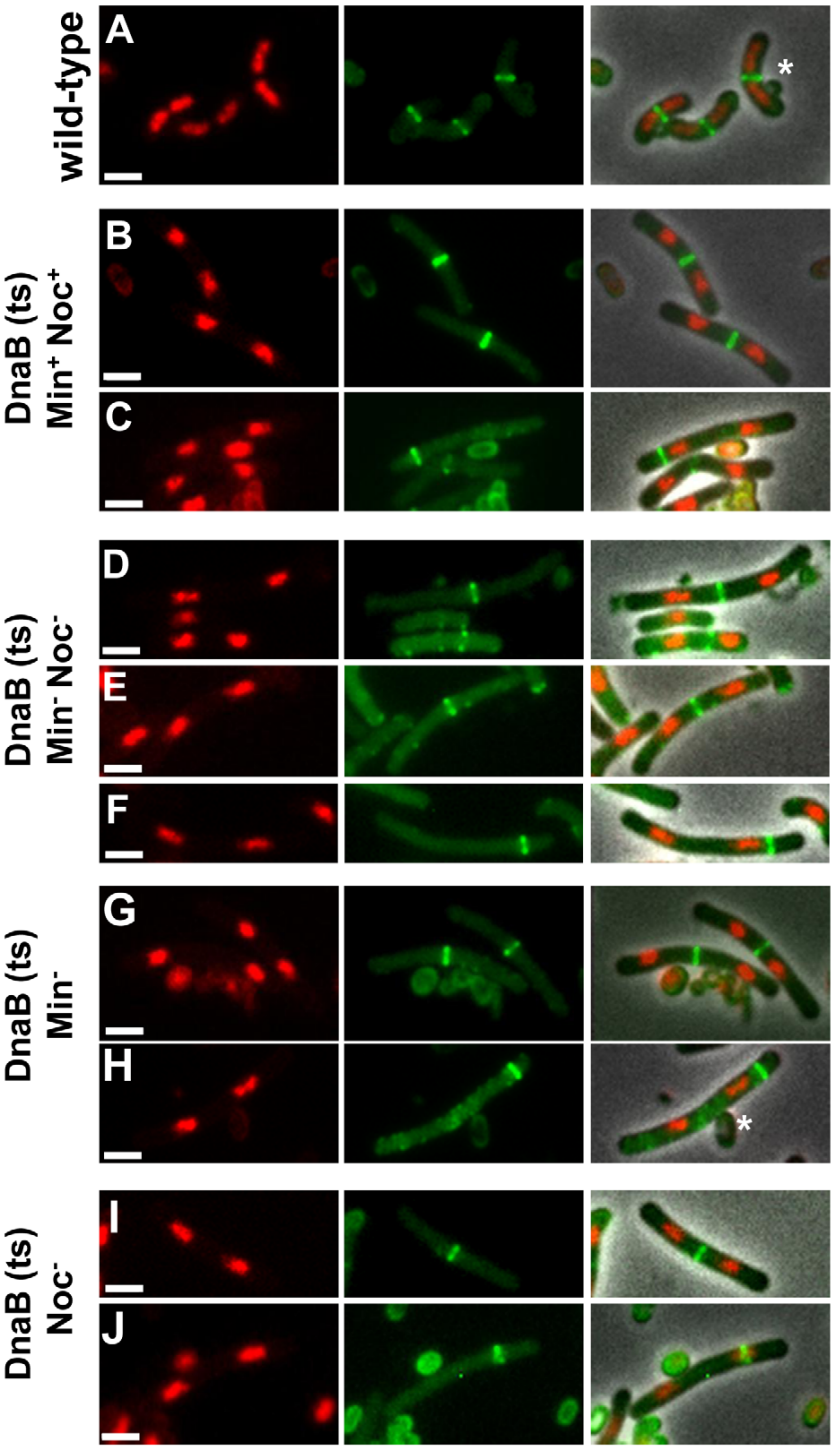
Z ring formation and positioning in cells with two separated nucleoids. (A) Co-visualization of Z rings and nucleoids in the wild-type control strain SU492 (DnaB^+^). These cells were outgrown as for Figure 4B. (B to J) Representative image of cells of the *dnaB* (ts) strains containing two separated nucleoids after induction of *ftsZ* expression (see Figure 4A) when both MinCD and Noc are present (SU671) (B and C), when both MinCD and Noc are absent (SU680) (D to F), when MinCD is absent (SU678) (G and H) and when Noc is absent (SU683) (I and J). (B, D, E, G, I) show a Z ring between the two nucleoids; (C, F and H) show a Z ring between the pole and one of the nucleoids and (J) shows a Z ring over one of the nucleoids. For more examples of cells with two nucleoids and a single Z ring refer to Figures S6 and S7. Images are DAPI (nucleoid) pseudo-coloured in red (left), FtsZ-YFP pseudo-coloured in green (middle) and phase-contrast fluorescence overlay (right). Stars denote autofluorescent spore coats. Scale bars are 2 µm.

For all four strains containing the *dnaB* (ts) mutation (Min^+^ Noc^+^, Min^−^ Noc^−^, Min^−^ and Noc^−^) we determined the number of cells that contained Z rings and the frequency of cells with two separated nucleoids containing a single Z ring (at 165 min; Figure 4A; Figure 5B). In Min^+^ Noc^+^ *dnaB* (ts) cells (SU671), the percentage of cells in the population with Z rings was 66%. More than half of these cells contained a single Z ring and two nucleoids (Figure 5B), with 94% being positioned between the nucleoids (Figure 5B and Figure 6B). The remaining 6% were positioned between the nucleoid and the pole (Figure 5B and Figure 6C; for more examples refer to Figure S6A–S6D). The proportion of cells with Z rings is reduced to 24% when both Min and Noc are absent (SU680; Figure 5B). This is consistent with the data above showing that the combined absence of the Min system and Noc results in a significant reduction in the frequency of cells with Z rings. Cells with two nucleoids and a single Z ring represented more than half (>50%) of the number of cells containing Z rings (Figure 5B). Remarkably, when Noc and the Min system are both absent (SU680) the vast majority (87%) of Z rings are still positioned between nucleoids (Figure 5B and Figure 6D and 6E); with the remaining 13% positioned between the nucleoid and the pole (Figure 5B and Figure 6F). More examples of cells with two nucleoids and a single Z ring in these cells are shown in Figure S6E–S6M). This surprising result demonstrates that even when there is no DNA (nucleoid occlusion) in the central region of the cell, no Noc and no Min system, almost all the Z rings that form are positioned between nucleoids rather than between the nucleoid and the pole.

**Figure 7:**
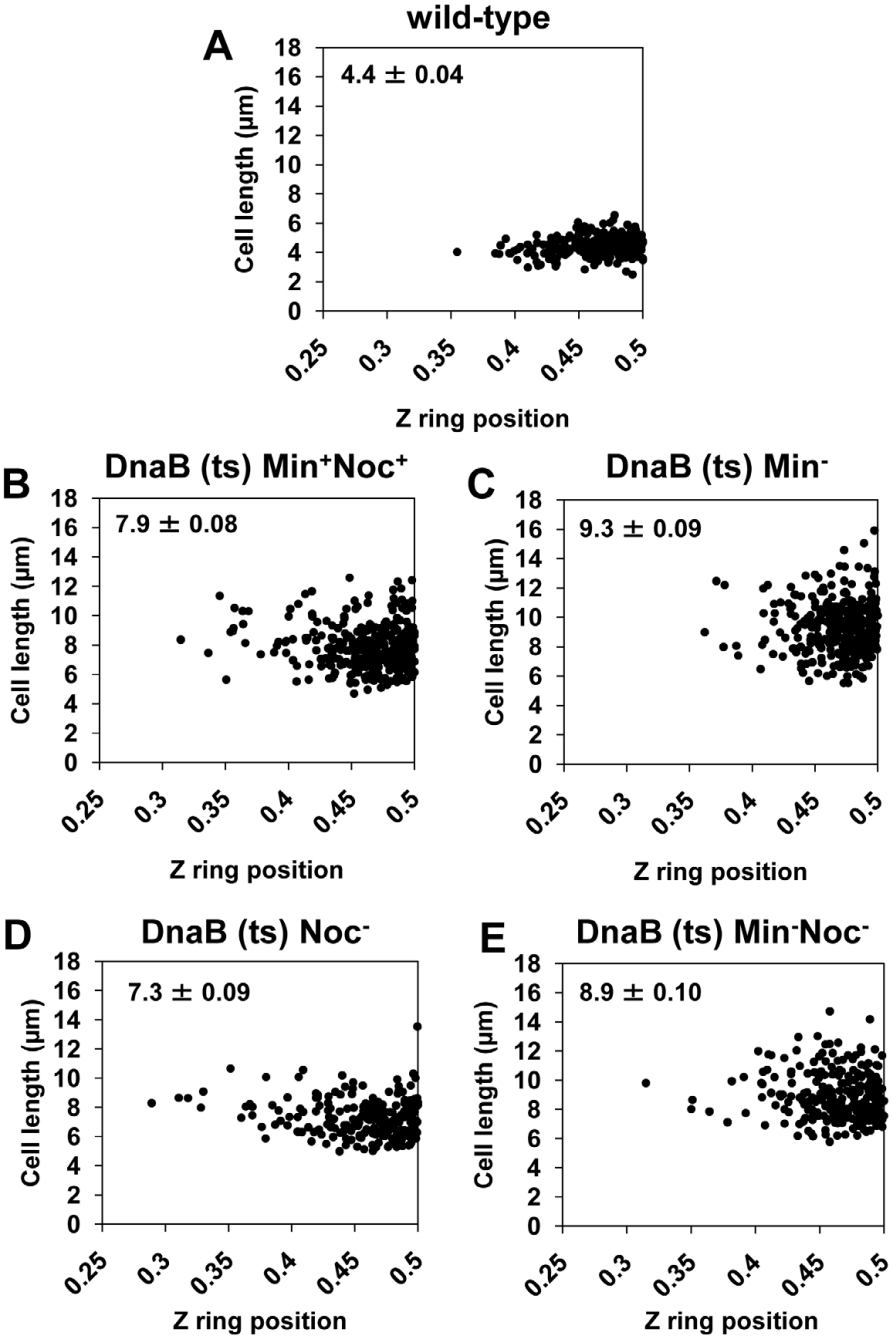
Z rings are positioned precisely between two separated nucleoids in the absence of Noc, Min, or both. The approach for this experiment is illustrated in Figure 4A. Spores of the *dnaB* (ts) strains containing P*_spac_-ftsZ* integrated at the *ftsZ* locus and P*_xyl_-ftsZ-yfp* at the *amyE* locus [strains SU671 (Min^+^, Noc^+^), SU678 (Min^−^), SU680 (Min^−^, Noc^−^), and SU683 (Noc^−^)] were germinated as described in the legend of Figure 4A. (A to E) Scatter plots demonstrating Z ring precision relative to cell length in the wild-type control (DnaB^+^, SU492) (A), in *dnaB* ts cells when Noc and MinCD are present (SU671) (B), in *dnaB* ts cells when MinCD is absent (SU678) (C), in *dnaB* ts cells when Noc is absent (SU683) (D) and in *dnaB* ts cells when both Noc and MinCD are absent (SU680) (E). Top left hand corner in each graph shows mean cell length of cells plotted (µm ± SEM). Over 200 cells were scored in each case.

We then determined whether the Z rings that formed in the double-mutant, *dnaB* (ts) cells are positioned at midcell with wild-type precision. As the control strain, we used wild-type outgrown spores expressing *ftsZ-yfp* (DnaB^+^; SU492) and outgrown for 105 min at 34°C and then shifted to the non-permissive temperature (48°C) for 30 min. Under these conditions the majority of cells (80%) had a single medially-located Z ring (Figure 6A), and 76% of these Z rings were positioned in the 0.45 to 0.5 region of the cell (Figure 7A). In Min^+^ Noc^+^ cells containing the *dna-1* mutation (SU671) 75% of the Z rings were positioned within the 0.45–0.5 region (Figure 7B). In Min^−^ Noc^−^ cells (SU683) 74% of Z rings were positioned within this wild-type range (Figure 7E). A statistical analysis was performed (Kolmogorov-Smirnov test) to compare the positioning of Z rings (including those outside the 0.45–0.50 range) that formed between two separated nucleoids in Min^+^Noc^+^ *dnaB* (ts) (SU671) and Min^−^ Noc^−^ *dnaB* (ts) (SU680) cells relative to that of the wild-type cells replicating their DNA normally (SU492, DnaB^+^). There was no statistical difference in the distribution of Z rings (p>0.05; data not shown) for both conditions relative to the wild-type control (SU492, DnaB^+^). These data strongly suggest that neither the Min system, Noc, or in fact any nucleoid occlusion is actually required for the placement of a Z ring precisely at midcell in *B. subtilis*.

We also examined the effect of single deletions of *noc* and *minCD* under the conditions of our experimental approach described above (that is, in a *dnaB* (ts) mutant background). The results were very similar to the double-mutant (Figure 5B; Figure 6G–6J). In both single mutants however, the proportion of cells with Z rings in the population was slightly higher than in the double-mutant [34% and 31% in Noc^−^ (SU683) and Min^−^ (SU678) cells, respectively compared to 24% for the double-mutant (SU680); Figure 5B]; as was the percentage of Z rings positioned between two nucleoids (92% and 94% for single mutants versus 87% for the double-mutant strain; see Figure 5B). More examples of cells with two nucleoids and a single Z ring are shown in Figure S7. In these single mutant *dnaB* (ts) cells, the remainder of the Z rings were located between the nucleoid and the pole in Min^−^ *dnaB* (ts) cells (Figure 6H) and over a nucleoid in Noc^−^ *dnaB* (ts) cells (see Figure 6J). Again, there was no statistical difference (p>0.05, Kolmogorov-Smirnov test; data not shown) between the position of Z rings (including those outside the 0.45–0.50 range) that formed between two separated nucleoids in Min^−^ (SU678) and Noc^−^ (SU683) *dnaB* (ts) strains relative to that of the wild-type replicating cells (SU492, DnaB^+^) (Figure 7C and 7D).

Under these conditions immunoblotting analysis showed essentially the same cellular level of FtsZ for all four *dna-1* strains, including the Min^+^ Noc^+^ strain, SU671 (data not shown). Thus the reduction in Z ring formation observed when Min and Noc or both are deleted from the *dna-1* strain (SU678, SU683 and SU680), is not likely to be due to decreased cellular levels of FtsZ. Interestingly however, we did observe approximately 30%–40% less FtsZ in all four *dna-1* strains at 49°C compared to wild-type (DnaB^+^) cells (SU5; data not shown). The reason for this is unclear.

As with the double-mutant cells, in most single-mutant cells with two separated nucleoids that did not contain Z rings, very faint FtsZ helical structures, dots and accumulations of FtsZ were observed (see Figure S8).

Collectively these results demonstrate that neither Min nor nucleoid occlusion is required for positioning the Z ring precisely at midcell in *B. subtilis*. Most remarkably, our data indicate that there is a substantial preference for Z rings to form precisely at midcell, as opposed to other DNA-free regions, in the absence of these systems. This strongly suggests the existence of an additional mechanism for identifying the cell centre in this organism. Thus the role of Min and nucleoid occlusion in Z ring positioning appears to be in ensuring efficient Z ring assembly specifically at the midcell site by preventing Z rings forming at inappropriate sites, as well as ensuring the overall efficiency of Z ring assembly.

### Z ring positioning does not correlate with the amount of DNA–free space at midcell

**Figure 8:**
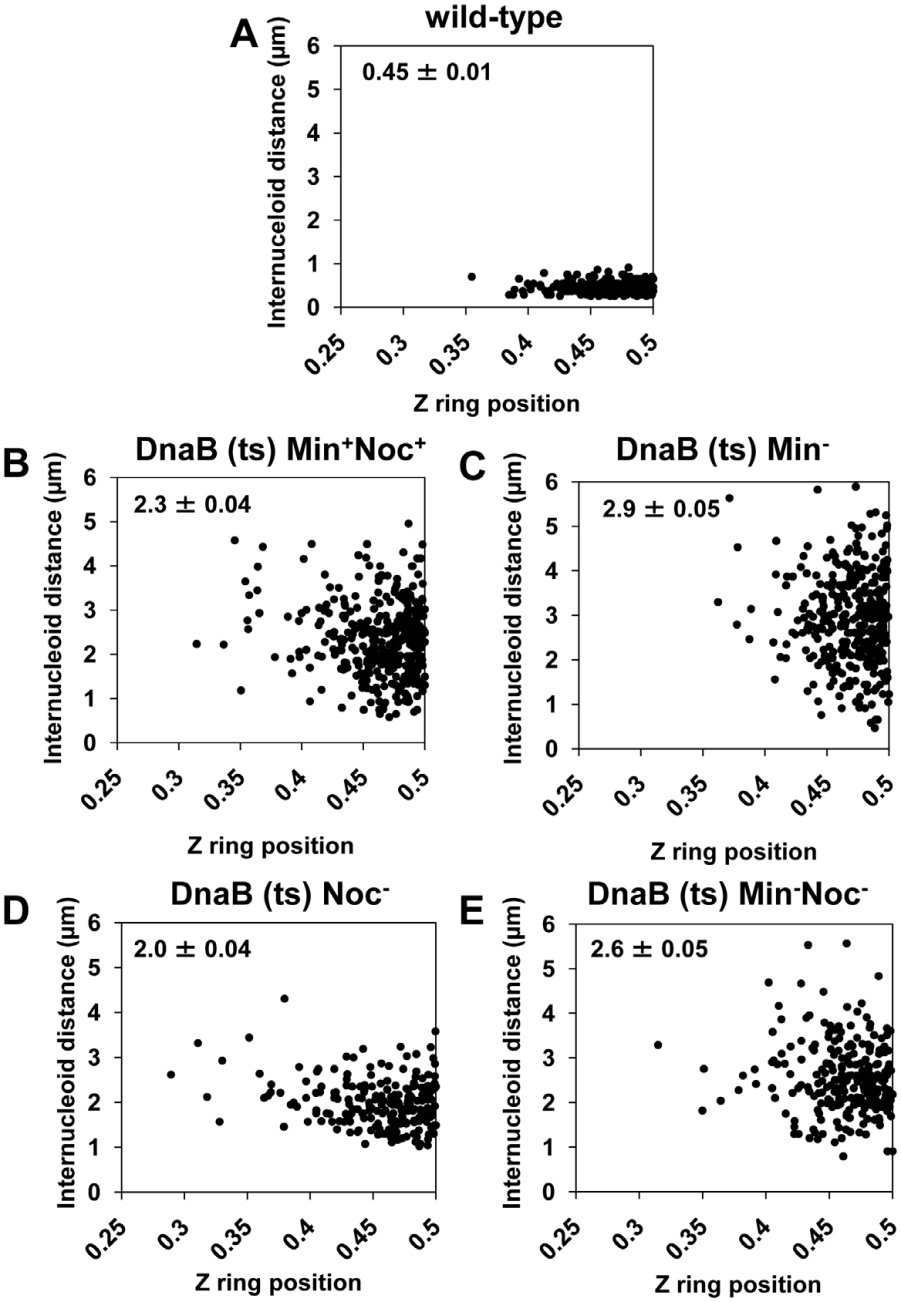
Midcell Z ring precision does not correlate with internucleoid distance. Cells were prepared as described in Figure 4A. (A to E) Scatter plots, using the *dnaB* (ts) cells as for Figure 7, showing the Z ring location relative to internucleoid distance in the wild-type control (DnaB^+^, SU492) (A), in *dnaB* (ts) cells when Noc and MinCD are present (SU671) (B), when MinCD is absent (SU678) (C), when Noc is absent (SU683) (D) and when both Noc and MinCD are absent (SU680) (E). Top left hand corner shows mean internucleoid distance (µm ± SEM). Over 200 cells were scored in each case.

Our data show that relieving nucleoid occlusion at midcell beyond normal wild-type limits and prior to Z ring assembly has no significant effect on the precise positioning of Z rings at the division site at midcell. In wild-type cells replicating DNA normally (DnaB^+^), Z rings were observed at midcell within a small DNA-free gap between segregating nucleoids (0.45±0.01 µm) - this corresponds to relief of nucleoid occlusion at midcell (see Figure 4B and Figure 5A). If nucleoid occlusion has a role in determining precisely where the Z ring will be placed between two segregating nucleoids, one might expect that as the internucleoid (DNA-free) gap at midcell increases, the precision of Z ring positioning at midcell would decrease. To test this idea, we determined whether there was any correlation between the amount of relief of nucleoid occlusion (i.e. size of the internucleoid space) and position of Z rings between two separated nucleoids. We used the same cells in Figure 6 - this is shown Figure 8. The scatter plots reveal no obvious relationship between the size of the internucleoid distance and precision with which a Z ring is positioned at the division site. Essentially, for all conditions tested [Min^+^Noc^+^ (SU671), Min^−^ (SU678), Noc^−^ (SU683), and Min^−^ Noc^−^ (SU680)] the majority of Z rings are clustered at positions 0.45–0.5, regardless of the internucleoid distance (Figure 8B–8E). A statistical analysis also showed that, while there was a very small negative correlative relationship between Z ring positioning and internucleoid distance (as internucleoid distance increases, midcell Z ring precision decreases) for all strains including the wild-type replicating cells (DnaB^+^, SU492; Figure 8A), this was not statistically significant (p>0.05; Kendall's tau coefficient and Spearman's rho coefficient; data not shown). Thus, Z ring positioning does not appear to correlate with the amount of relief of nucleoid occlusion (i.e. size of the internucleoid gap) at midcell. This convincing result establishes that nucleoid occlusion (or its relief) is a weak determinant, if at all, in defining the position of the division site in *B. subtilis*. Thus, while nucleoid occlusion plays a dominant role in determining whether the Z ring forms at midcell or not, it does not identify the division site, nor is it responsible for the precision of Z ring positioning at this site.

## Discussion

Positioning of the division site at midcell in rod-shaped bacteria such as *B. subtilis* and *E. coli* is currently believed to result primarily from the combined action of Noc and the Min system [1]–[3], [8]. We now show that, remarkably, the Z ring can be positioned precisely at the division site in *B. subtilis* in the complete absence of these two systems. We further demonstrate that in the absence of both the Min system and all nucleoid occlusion, there is a substantial preference for Z rings to form at midcell between two nucleoids and with wild-type precision, rather than in any other DNA-free region of the cell. Moreover, the actual position of Z rings that formed between nucleoids showed no correlation with the size of the DNA-free gap between nucleoids. Our results reveal the existence of an additional mechanism that identifies the division site at midcell independently of the Min system and nucleoid occlusion.

While we cannot rule out the possibility that the results obtained here using outgrowing spores cannot be extrapolated to reflect the situation in vegetatively-growing cells, we believe this is highly unlikely since this system for *B. subtilis* has been shown in several studies to accurately reflect both the molecular events of the cell cycle and their spatio-temporal regulation in vegetative growth [35], [51], [52]. Furthermore, data obtained from vegetatively-growing cells here is entirely consistent with that obtained with outgrown spores.

### A mechanism independent of Min and nucleoid occlusion identifies the division site in *B. subtilis*

**Figure 9:**
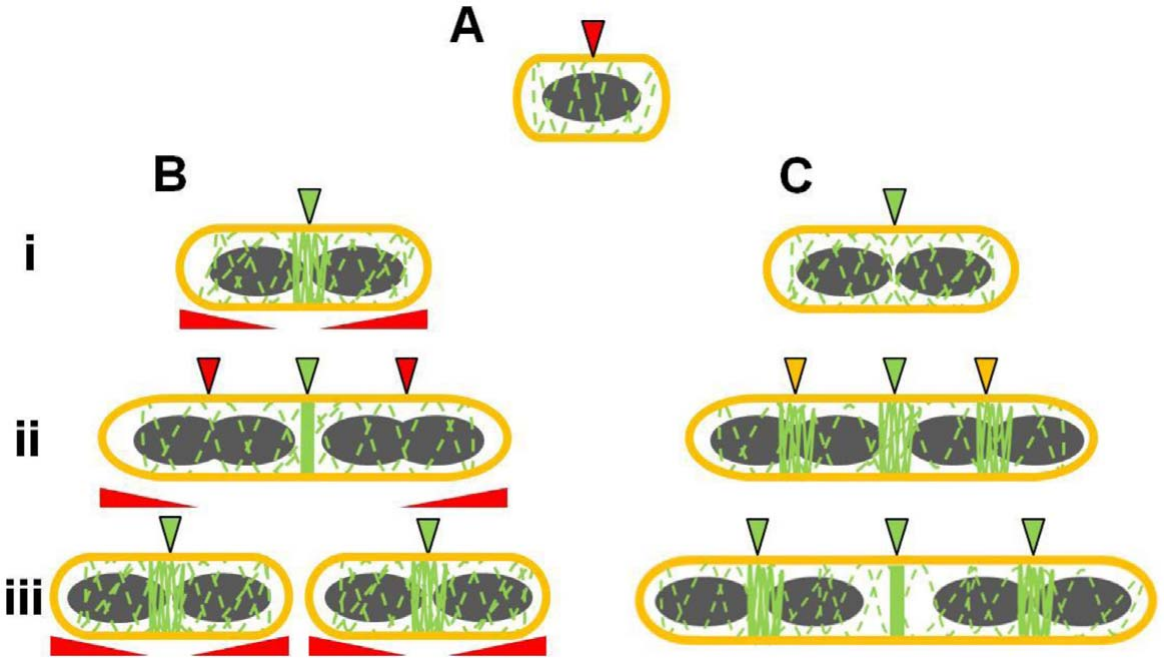
Model for the role of Noc and Min in ensuring efficient utilization of the division site in *B. subtilis*. (A) Early in the cell cycle, possibly upon completion of the initiation phase of DNA replication (see Moriya *et al*, 2010) a midcell-defining factor marks the position of the future division site at which the Z ring will assemble. At this stage, midcell becomes competent for Z ring assembly. However the early utilization of this division site by the Z ring is blocked by nucleoid occlusion (demonstrated by the red arrow). (Bi) In wild-type cells, the midcell site becomes unmasked (in green) upon segregation of the bulk of the replicated chromosomes (shown in grey). This causes Noc and other nucleoid occlusion factors to clear the central region of the cell. (Bii) As the cells elongate, other division sites competent for Z ring assembly become available at the cell quarters. These sites are masked (in red) by the combined activity of Noc (and possibly other nucleoid occlusion factors), and Min that acts at a distance from the pole, allowing FtsZ to concentrate to the midcell division site only. The red elongated triangles below each cell correspond to the position and concentration of Min (higher at the poles). (Biii) The quarter (potential) division sites only become available after Z ring constriction is initiated at the first (midcell) site, allowing separated daughter cells to initiate a new division cycle. (Ci) In the absence of Noc and MinCD, despite midcell being competent for Z ring assembly, utilization of this site is delayed due to the titration of FtsZ to the cell poles, and the inability of FtsZ to reach a high enough threshold concentration at midcell to form a ring. (Cii) As the cells grow, FtsZ can eventually start to accumulate at midcell, but also at other division sites that would normally be blocked by Noc and MinCD (in yellow). (Ciii) Z ring assembly at the medial division site (midcell) occurs very late, well after wild-type cells would have already completed a round of division, causing a severe delay in cell division. The dashed green lines along the length of the cell indicate the constant dynamic movement of FtsZ throughout the cell.

It has already been shown convincingly that both the Min system and nucleoid occlusion play important roles in positioning the Z ring in both *B. subtilis* and *E. coli* [1]–[3], [31], [53]. Our data are indeed consistent with this role and show that these two factors are absolutely required for the efficient formation of Z rings in *B. subtilis*, as well as for ensuring that Z rings form at midcell and not at other positions. However, several observations made here lead us to propose a model in which nucleoid occlusion and Min do not identify the correct division site at midcell *per se*, but ensure that the Z ring forms there and only there, at the right time in the cell cycle. This is illustrated in Figure 9.

The distinct preference for Z rings to assemble at midcell, instead of the poles, in the absence of Min and Noc is entirely consistent with a previous study showing that in minimal medium almost all Z rings are positioned at midcell in the absence of *minCD* [54], and with a more recent study by Pogliano and co-workers that supports a primary role for the Min system in preventing polar (or secondary) Z ring assembly at new cell poles [28]. In doing so, it prevents FtsZ from being titrated to polar sites, thus contributing to the correct timing of cell division at midcell [28]. Thus, rather than acting to provide positional information regarding the actual site of Z ring assembly, we propose that the Min system in *B. subtilis* acts to ensure efficient and timely usage of this site. The significant decrease in Z ring formation when *minCD* was deleted in cells in which two replicated nucleoids were allowed to separate is consistent with this idea.

Rudner and co-workers have recently shown that even in the absence of Noc, other nucleoid occlusion factors appear to prevent Z ring assembly over DNA [45]. Our findings support the existence of additional nucleoid occlusion factors since we rarely observed Z rings over nucleoids in the absence of Noc in all experiments. Our data however strongly argue against the involvement of any of these other nucleoid occlusion factors in defining the site for Z ring assembly at midcell in *B. subtilis* (see Figure 9).

How then does a bacterial cell find its middle? One possibility is that a signal or structure marks the midcell position for Z ring assembly independently of nucleoid occlusion and the Min system [1], [37], [55]–[58]. This could be a protein or a physical change in the conformation or type of membrane lipids at midcell [59]–[61]. The “Ready-Set-Go” model linking identification (potentiation) of the midcell division site for Z ring formation with the progress of the initiation phase of DNA replication proposes such a signal [38]. The recent discovery of two proteins SsgA and SsgB that are required to target FtsZ to division sites during sporulation in *Streptomyces coelicolor* [40] highlights the presence in bacteria of factors independent of the Min system and nucleoid occlusion that identify the division site. Our data strongly support this possibility in *B. subtilis*, and the idea of a midcell marker for Z ring assembly definitely merits further investigation.

### A role for Noc and the Min system in ensuring efficient midcell Z ring assembly at a single pre-determined division site

The dramatic delay in midcell Z ring assembly observed in outgrown cells in the absence of both *noc* and *minCD* suggests an inability of these cells to restrict FtsZ accumulation to a single division site, resulting in the accumulation of FtsZ at second division sites, as well as at the cell poles. Consistent with this idea, we show that FtsZ overproduction partially restores both the efficiency (see also [29]) and the timing of midcell Z ring formation. This is a similar conclusion to that described recently for *min* mutants by Pogliano and co-workers [28].

To test whether Min and Noc function to maximise Z ring formation at midcell solely by preventing titration of FtsZ to second division sites, we attempted to determine the effect of different growth rates on the efficiency of Z ring assembly specifically at midcell in Noc^−^ Min^−^ cells. While it was not possible to interpret the results fully, the data did suggest that at slower growth rates when there are less division sites, the efficiency of midcell Z rings increases in the double-mutant cell population. However, since the average length of *noc minCD* double-mutant cells was still significantly longer than those of the wild type (data not shown), this does not fully explain the phenotype of the double-mutant cells. We therefore propose that even in cells that are growing slowly enough such that they contain only one division site competent for Z ring assembly, these two factors have an additional role in concentrating FtsZ to the central region of the cell, to allow a Z ring to form there. The possibility that Min proteins act away from the pole towards midcell in *B. subtilis* to prevent accumulation of FtsZ in non-central regions of the cell has been raised previously [29]. Two very recent studies [28], [62] have suggested that the cell poles are potential division sites in *B. subtilis* in the sense that Min appears to prevent the re-formation of the divisome at the nascent pole during septation. In this respect, potential division sites refer to midcell, future division sites at the quarter positions, and to cell poles. In other words, we propose that Min and Noc act together to prevent Z rings forming at all these potential division sites.

During spore outgrowth in the absence of Noc and Min we observed very early accumulation of FtsZ at several division sites. Since Noc can prevent a significant number of Z rings forming precisely at midcell even without completion of the initiation stage of DNA replication [36]–[38] it seems likely that when Noc is absent, FtsZ can start to accumulate at these division sites very early in the round of replication, even before the elongation phase, and Noc plays a role in hindering midcell Z ring assembly over unreplicated DNA [38]. This role for Noc is consistent with recent data showing that it also appears to act as a subtle timing device for cell division in cells replicating DNA normally [34]. Recent evidence indicates the existence of other Noc-independent nucleoid occlusion factors that prevent premature Z ring assembly over DNA, at least during the elongation phase of replication [45].

Interestingly, we observed that, although delayed, the first Z rings that form in double-mutant outgrown cells replicating DNA normally were predominantly located at the medial division site (older division site; over 60%). Given that these longer-than-normal double-mutant cells already contained multiple chromosomes at the time of Z ring formation (and thus more than one division site) it appears that some factor is attracting Z ring assembly to the oldest (first) division site at the cell centre. This could occur through a putative positive signal for Z ring assembly (mentioned above) that is present at the first division site earlier than subsequent division sites, causing accumulation of FtsZ, and finally a functional Z ring at midcell. However in the absence of Min and Noc, very soon after this (at or after initiation of DNA replication) the second division sites become available and FtsZ starts to accumulate also at these sites (Figure 9). Since FtsZ accumulates at midcell earlier than subsequent division sites, there is always more FtsZ here than at other sites. The perhaps slower but constant accumulation of FtsZ at the midcell site in the absence of Noc and MinCD enables the concentration of FtsZ to reach the required level to form a Z ring first. We cannot exclude the possibility that the Min system and Noc function to ensure the efficient and rapid transitioning between the different helical-like states adopted by FtsZ in normal wild-type cells during the cell cycle, as described by recent models for Z ring assembly [42], [43]. This is an attractive idea and warrants further investigation.

## Materials and Methods

### Bacterial strains and growth conditions

*B. subtilis* strains are listed in Table 1. The transformations to obtain strains produced as part of this work are also described in Table 1. All strains containing a *minCD::cat* knockout cassette originate from SU561. In SU561 only the first 22 amino acids of *minC* and the final 23 amino acids of *minD* remain intact. The remaining portion of these genes was replaced by the *cat* resistance gene. All of these strains showed a *minCD*-deletion phenotype with cells having a 30–40% longer average cell length relative to wild-type and the production of minicells. All strains containing a *noc::tet* knock-out cassette strain originate from the *noc::tet*-containing strain 1284 [29]. Strain SU661 was obtained by congression of SU5 with SU46 chromosomal DNA. The presence of the *dna-1* mutation was confirmed by sequencing the *dna-1* open reading frame. Transformations involving the *dna-1* mutation were also confirmed to be temperature sensitive for DNA replication.

Bacteria were grown vegetatively in liquid PAB (antibiotic medium 3, Difco, USA) or semi-solid TBAB (tryptose blood agar base, Difco, USA) at 30°C or 37°C. Thymine where required was added to a final concentration of 20 µg ml^−1^ unless otherwise indicated. Spectinomycin (40–60 µg ml^−1^), tetracycline (10 µg ml^−1^), chloramphenicol (5 µg ml^−1^), erythromycin (0.5 µg ml^−1^), neomycin (1–2 µg ml^−1^) and phleomycin (2 µg ml^−1^) were added as appropriate to TBAB plates or overnight cultures. To induce expression of *amyE::*P*_xyl_*-*ftsZ-yfp*, 0.01% (w/v) xylose was included in the medium. To induce expression of *ftsZ::P_spac_-ftsZ*, 1 mM IPTG was included in the medium and for *amyE::P_spachy_-ftsZ*, different concentrations of IPTG were added as applicable.

Spores were prepared and harvested as described previously [63]. Spore germination and outgrowth was performed with 2×10^8^ spores ml^−1^ in PAB at 34°C [36], [37]. Antibiotic selection was not applied during spore outgrowth. For outgrowth in the experimental approach outlined in Figure 4A, spores of the *dnaB* (ts) strains SU671 (Min^+^, Noc^+^), SU678 (Min^−^), SU680 (Min^−^, Noc^−^), and SU683 (Noc^−^) were germinated in 3 ml of GMD [37] for 105 min at 34°C and then transferred to 48°C to prevent re-initiation of DNA replication and allow separation of replicated nucleoids. At 135 min (still at 48°C) 1 ml of culture was removed for visualization and measurement of the extent of DNA separation. To the remaining 2 ml of culture specific volumes of IPTG and xylose were added to a final concentration of 1 mM and 0.01%, respectively, to induce *ftsZ* expression at the level of P*_spac_ ftsZ* and P*_xyl_ ftsZ-yfp*, respectively. This 2 ml culture continued incubation at 48°C for another 30 min (165 min in total) after which cells were collected for visualization of Z rings and nucleoids. Wild-type control strain SU492 (DnaB^+^) was outgrown at 34°C in GMD with 0.01% xylose for 105 min and then transferred to 48°C for 30 min, to allow visualization of the first Z rings formed during spore outgrowth. For all other outgrowth experiments, spores were incubated at 34°C with shaking for the time indicated.

### Ethanol-fixation of cells

Cells (1 mL) were collected and pelleted in a microcentrifuge. The pellet was resuspended in 300 µL PBS and cells were fixed with the addition of 700 µL cold ethanol (95% (v/v), 4°C). Cells were fixed at 4°C (24–48 h). The suspension was pelleted in a microcentrifuge and washed twice by resuspension and centrifugation in 200 µL PBS. The pellet was resuspended in 200 µL PBS. The fixed, washed cells (10 µL) were transferred to poly-L-lysine treated multi-well slides (ICN Biochemicals) and incubated at room temperature for 5 min. The excess liquid was aspirated off and the wells were washed once with PBS. The slide was mounted in PBS containing glycerol (50%) and the edges of the coverslip were sealed with nail polish.

### Fluorescence microscopy

Immunofluorescence microscopy was performed using affinity-purified rabbit anti-FtsZ antibodies (raised against *B. subtilis* FtsZ) and Alexa 488-conjugated secondary antibodies (Molecular Probes, Invitrogen, USA) as described previously [42]. Live cells were visualized on 2% (w/v) agarose pads using Gene Frame (AB Genes) to create a flat surface on the glass slide. DAPI was added at a final concentration of 0.4 µg ml^−1^ before placing the cells on the agarose pad. The same procedure was performed for co-visualization of the nucleoid and Z ring in live cells. All images were taken on a Zeiss Axioplan 2 fluorescence microscope equipped with a Plan ApoChromat (100×, NA 1.4; Zeiss) objective lens or a UPlan Fluorite phase-contrast objective (oil immersion objective; 100×; NA 1.3; Olympus) and a Zeiss AxioCam MRm cooled CCD camera. The light source was a 100 W high pressure mercury lamp passed through the following filters: for visualising Alexa 488 (Filter set 09, Zeiss), for visualising DAPI (Filter set 02, Zeiss; 365), and for visualising YFP (Filter set 41029, Chroma Technology). Image processing was performed using AxioVision 4.5 or 4.6 software (Zeiss) and scoring of these images was performed using AxioVision software. Image quality was improved by applying the cubic spline interpolation algorithm (to reduce pixelation) available on AxioVision software. Images were exported as TIFF files.

### Measurement of cell lengths, Z ring positioning, internucleoid distances, and calculation of statistics

Pixel to µm scaling for images collected by an AxioCam MRm camera were used to calculate cell lengths and other cell measurements from digital images. Numerical values for each measurement were exported from the AxioVision software as a text file, and imported into Excel (Microsoft) for final analysis. Excel was used to calculate mean, standard deviation, standard error of the mean (SEM) and the number of cells counted (n) for each data set, as well as for the production of scatter plots and histograms.

We scored FtsZ localization as a ring if it was a transverse band oriented perpendicularly to the long-axis of the cell. FtsZ localization patterns that showed a band oriented diagonally to the long-axis of the cell were not scored as Z rings, and were not included in the Z position analysis.

Z ring position was determined by measuring the distance from the Z ring to the closest cell pole divided by the total cell length, with 0.5 being precisely midcell. A Z ring was considered to be at midcell if it was positioned within the range of 0.45–0.50, where essentially all Z rings formed in the wild-type background under the same conditions. The internucleoid distance in cells with two nucleoids was determined by measuring the distance between two regions of DAPI-stained nucleoids, from edge-to-edge. For all measurements, cells were considered for analysis and measurement only if completely contained within the field of view. For this reason, the average cell length of the *noc minCD* double-mutant cells should be considered an estimate only.

Statistical analyses were carried out using SPSS software version 17 (IBM). Statistical analyses included the non-parametric “Kolmogorov-Smirnov” test, the “Kendall's tau coefficient” and “Spearman's rho coefficient”. The “Kolmogorov -Smirnov” test was used to compare the precision of midcell Z ring positioning in wild-type and mutant strains. The “Kendall's tau coefficient” and “Spearman's rho coefficient” were used to identify correlations between the size of internucleoid distances and Z ring positioning. All statistics were performed using a 95% confidence interval, where p<0.05 indicates a statistically significant difference between the comparisons made.

### Western blot analysis

*B. subtilis* cells (10 mL of exponentially-growing vegetative cells; OD_600_ 0.4–0.6) cell lysates were prepared as described previously (Harry et al. 1993). Sample volumes representing an equal loading of protein were loaded for SDS-PAGE into pre-cast NuPAGE 4–12% Bis-Tris gels (Invitrogen). The iBlot (Invitrogen) semi-dry transfer system was used, according to the manufacturer's instructions. After Western transfer, the blot was incubated in blocking solution [PBS+5% (w/v) skim milk powder] for 2 h at RT with rocking. The blot was then incubated with a primary FtsZ antibody (raised in rabbit), diluted 1 in 10,000 in blocking solution, for 2 h. Next, the blot was washed three times in blocking solution containing 0.05% (v/v) Tween 20 and incubated with HRP-conjugated secondary antibody (Promega) diluted 1 in 2,500 in PBS for 1 h. Band detection was carried out using enhanced chemiluminescence reagents (GE Healthcare) according to the manufacturer's instructions, and the ChemiDoc XRS_+_ imaging system (Bio-Rad). Band intensities were analysed by densitometry using Quantity One software (version 4.6.1, Bio-Rad).

To ensure quantification of protein band intensities within a linear range, a standard curve of was obtained using 2-fold serial dilutions of protein samples. Only bands within the linear range of the standard curve were quantified.

## Supporting Information

Figure S1 Cell length distribution of vegetatively-growing wild-type and *noc minCD* double-mutant strains at 30°C and 37°C. Cells of the wild-type (SU5) and the double-mutant (SU681) were grown in PAB at 30°C or 37°C, collected at the mid-exponential phase of vegetative growth and prepared for ethanol fixation to examine cell lengths or prepared for immunofluorescence to visualize FtsZ. (A and B) Cell-length distribution of ethanol-fixed wild-type (black bars) and double-mutant cells (grey bars) grown at 30°C (A) and 37°C (B). (C to F) FtsZ localization in wild-type (C and D) and double-mutant (E and F) cells grown at 30°C (C and E) or 37°C (D and F) and prepared for immunofluorescence. Images are phase contrast (left), and FtsZ immunofluorescence (right). Scale bars are 5 µm.
(TIF)

Figure S2 FtsZ overproduction in vegetatively-growing wild-type and *noc minCD* double-mutant cells grown at 30°C. Wild-type (SU558) and the double-mutant (SU685) cells containing P*_spachy_-ftsZ* integrated at the *amyE* locus were grown in PAB at 30°C, with increasing concentrations of IPTG and collected during mid-exponential phase. (A) Cell length distribution of ethanol-fixed FtsZ-overproducing cells of the wild-type (dark grey bars) and the double-mutant (light grey bars) strains at 30°C. Error bars are µm ± SEM. (B and C) FtsZ localization in the wild-type grown in the absence (B) or presence of 0.05 mM IPTG (C). (D) FtsZ localization in the double-mutant strain grown in the presence of IPTG (0.05 mM). Images are phase contrast (upper panel), and FtsZ immunofluorescence (lower panel). Scale bars are 2 µm. (E) Western analysis of FtsZ levels in wild-type and double-mutant vegetatively-growing cells overproducing FtsZ at 30°C. Lanes were loaded with whole cell lysates of exponentially-growing cells including: (1) the wild-type strain not containing P*_spachy_*-*ftsZ* (SU5) as a control; (2) the double-mutant strain not containing P*_spachy_-ftsZ* (SU681); (3) the wild-type strain containing P*_spachy_-ftsZ*, in the absence of IPTG (SU558); (4) the wild-type strain containing P*_spachy_-ftsZ*, in the presence of 0.05 mM IPTG (SU558); (5) the double-mutant strain containing P*_spachy_-ftsZ*, in the absence of IPTG (SU685) and (6) the double-mutant strain containing P*_spachy_-ftsZ*, in the presence of 0.05 mM IPTG (SU685). Estimated molecular mass of the FtsZ band is 40 kDa. Numerical values under the bands show the mean (± standard deviation) intensity of the bands relative to the wild-type strain not containing P*_spachy_*-*ftsZ* (SU5) (n = 2). FtsZ overproduction with 0.05 mM IPTG had very little effect on cell length in a wild-type background (SU558, P*_spachy_-ftsZ*) at 30°C. Occasionally minicells (data not shown) as well as a slightly higher proportion of cells containing more than one Z ring (compare Figure S2B to S2C) were observed. In the wild-type (SU558) and double-mutant (SU685) strains, growth in PAB at 30°C supplemented with 0.1 mM IPTG resulted in a detrimental effect on cell length (Figure S2A) and growth rate (judged by growth curve; data not shown).
(TIF)

Figure S3 FtsZ overproduction in vegetatively-growing wild-type cells and *noc minCD* double-mutant cells at 37°C. Wild-type (SU558) and the double-mutant (SU685) cells containing P*_spachy_-ftsZ* integrated at the *amyE* locus were grown in PAB at 37°C, with increasing concentrations of IPTG, collected during mid-exponential phase and fixed with ethanol. (A) Cell length distribution of FtsZ-overproducing cells of the wild-type (dark grey bars) and the double-mutant (light grey bars) at 37°C. Error bars are µm ± SEM. (B to D) Representative phase-contrast images of double mutant cells grown in absence of IPTG (B), with 0.01 mM IPTG (C) or with 0.05 mM IPTG (D). Scale bars are 5 µm. FtsZ overproduction with 0.05 mM IPTG had very little effect on cell length in a wild-type background (SU558, P*_spachy_-ftsZ*) at 37°C. In the wild-type (SU558) and double-mutant (SU685) strain, growth in PAB at 37°C supplemented with 0.1 mM IPTG resulted in a detrimental effect on cell length (Figure S3A) and growth rate (judged by growth curve; data not shown).
(TIF)

Figure S4 Overproduction of FtsZ using FtsZ-YFP in live wild-type and *noc minCD* double-mutant cells. Cells of the wild-type (SU492) and the double-mutant (SU663) strain containing P*_xyl_-ftsZ-yfp* at the *amyE* locus were grown in PAB at 30°C, with various concentrations of xylose. Samples were collected during mid-exponential phase. (A) Cell lengths in ethanol-fixed wild-type (dark grey bars) and the double-mutant (light grey bars) cells during overproduction of FtsZ-YFP with increasing concentrations of xylose. Error bars are µm ± SEM. (B and C) FtsZ-YFP localization in live wild-type (B) and double-mutant cells (C) overproducing FtsZ using FtsZ-YFP (0.5% xylose) at 30°C. Images (left to right) are DAPI pseudo-coloured in red, FtsZ-YFP pseudo-coloured in green, and phase-contrast fluorescence overlay. Scale bars are 2 µm. Carets point to accumulations of FtsZ and stars denote minicells. FtsZ overproduction with 0.5% xylose had very little effect on cell length in a wild-type background (SU492). Occasionally minicells were observed. (D) Western analysis of total cellular FtsZ levels (both FtsZ-YFP and native FtsZ) in the double-mutant and the wild-type vegetatively-growing cells grown in the absence or presence of xylose (0.5%). Lanes contain: (1) the wild-type strain not containing P*_xyl_*-*ftsZ-yfp* (SU5) as a control; (2) the double-mutant strain not containing P*_xyl_*-*ftsZ-yfp* (SU681); (3) SU492 (P*_xyl_*-*ftsZ-yfp*) with no xylose; (4) SU492 (P*_xyl_*-*ftsZ-yfp*) with 0.5% xylose; (5) SU663 (*minCD noc* P*_xyl_*-*ftsZ-yfp*) in the absence of xylose and (6) SU663 (*minCD noc* P*_xyl_*-*ftsZ-yfp*) with 0.5% xylose. Estimated molecular mass of the bands is 40 kDa and 67 kDa, for FtsZ and FtsZ-YFP respectively. Numerical values under the bands shows the mean (± standard deviation) intensity of the FtsZ-containing bands relative to the wild-type strain not containing P*_xyl_*-*ftsZ-yfp* (SU5) (n = 2).
(TIF)

Figure S5 Z ring formation during spore outgrowth in *dnaB* (ts) cells is dependent on the addition of IPTG (and xylose) to the medium. Spores of *dnaB* (ts) strains containing P*_spac_-ftsZ* integrated at the *ftsZ* locus and P*_xyl_-ftsZ-yfp* at the *amyE* locus [SU671 (Min^+^, Noc^+^) SU678 (Min^−^), SU680 (Min^−^, Noc^−^) and SU683 (Noc^−^)] were outgrown at 34°C in PAB with (A to D) or without (E to H) 0.01% xylose and 1 mM IPTG, and collected for immunofluorescence at 150 min of spore outgrowth. Representative images of cells when Noc and MinCD are present (SU671) (A and E), when Noc is absent (SU683) (B and F), when MinCD is absent (SU678) (C and G) and when both Noc and MinCD are absent (SU680) (D and H). Images are phase contrast (left), and FtsZ immunofluorescence (right). Scale bars are 2 µm.
(TIF)

Figure S6 Z ring formation and positioning during spore outgrown in *dnaB* (ts) cells with two separated nucleoids that contain or lack both MinCD and Noc. See Figure 4A, for diagram, of experimental approach. Spores of the *dnaB* (ts) strains containing *ftsZ::P_spac_-ftsZ* and *amyE::P_xyl_-ftsZ-yfp* [SU671 (Min^+^, Noc^+^), SU680 (Min^−^, Noc^−^)] were germinated as described in the legend of Figure 4A. (A to D) Z ring localization in live cells with two nucleoids in cells containing both Noc and MinCD (SU671). (E to M) Z ring localization in live cells with two nucleoids in cells lacking both Noc and MinCD (SU680). (A to C and E to K) Z rings localizing between the two nucleoids. (D, L and M) Z rings localizing between the nucleoid and pole. Images (left to right) are DAPI pseudo-coloured in red, FtsZ-YFP pseudo-coloured in green and phase-contrast fluorescence overlay. Scale bars are 2 µm.
(TIF)

Figure S7 Z ring formation and positioning during spore outgrown in *dnaB* (ts) cells with two separated nucleoids that lack either MinCD or Noc. See Figure 4A, for diagram, of experimental approach. Spores of the *dnaB* (ts) strains containing *ftsZ::P_spac_-ftsZ* and *amyE::P_xyl_-ftsZ-yfp* [SU678 (Min^−^), SU680, SU683 (Noc^−^)] were germinated as described in the legend of Figure 4A. (A to F) Z ring localization in live cells with two separated nucleoids lacking MinCD. (G to K) Z ring localization in live cells with two separated nucleoids lacking Noc. (A to D and G to I) Z rings localizing between the two nucleoids. (E and F) Z rings localizing between the nucleoid and pole. (J and K) Z rings localizing between the two nucleoids (J) and Z rings localizing over one of the nucleoids (K). Images (left to right) are DAPI pseudo-coloured in red, FtsZ-YFP pseudo-coloured in green and phase-contrast fluorescence overlay. Scale bars are 2 µm.
(TIF)

Figure S8 Distinct accumulations of FtsZ in outgrown *dnaB* (*ts*) cells with two separated nucleoids. See Figure 4A, for diagram, of experimental approach. Spores of the *dnaB* (ts) strains containing *ftsZ::P_spac_-ftsZ* and *amyE::P_xyl_-ftsZ-yfp* [SU671 (Min^+^, Noc^+^) SU678 (Min^−^), SU680 (Min^−^, Noc^−^) and SU683 (Noc^−^)] were germinated as described in the legend of Figure 4A. (A to H) FtsZ localizations in cells containing both MinCD and Noc (A), in cells lacking both MinCD and Noc (B to D), in cells lacking MinCD (E and F), and cells lacking Noc (G and H). Images are DAPI pseudo-coloured in red (left), FtsZ-YFP pseudo-coloured in green (middle) and phase-contrast overlay (right). Carets point to likely helical-like patterns which are magnified in (F) and (H). Small, white arrowheads point to distinct foci or other less distinct accumulations of FtsZ. Scale bars are 2 µm.
(TIF)
