# Supplementary figures and images for: The Min System and Nucleoid Occlusion Are Not Required for Identifying the Division Site in Bacillus subtilis but Ensure Its Efficient Utilization
Source: PLoS Genet. 2012 Mar 22;8(3):e1002561. doi: 10.1371/journal.pgen.1002561 (PMC3310732; doi:10.1371/journal.pgen.1002561)

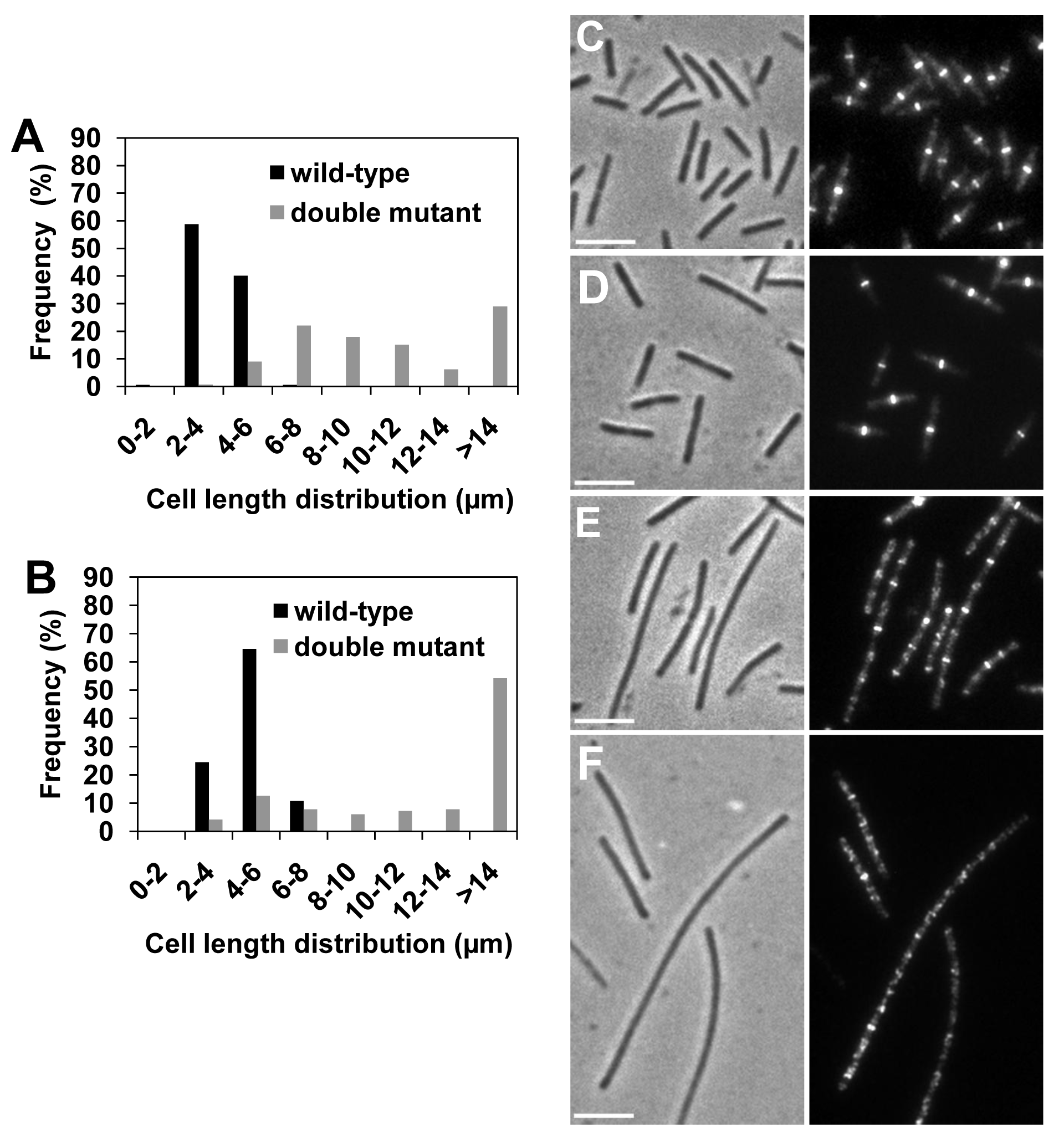

Supplement: Figure S1 — Cell length distribution of vegetatively-growing wild-type and noc minCD double-mutant strains at 30°C and 37°C. Cells of the wild-type (SU5) and the double-mutant (SU681) were grown in PAB at 30°C or 37°C, collected at the mid-exponential phase of vegetative growth and prepared for ethanol fixation to examine cell lengths or prepared for immunofluorescence to visualize FtsZ. (A and B) Cell-length distribution of ethanol-fixed wild-type (black bars) and double-mutant cells (grey bars) grown at 30°C (A) and 37°C (B). (C to F) FtsZ localization in wild-type (C and D) and double-mutant (E and F) cells grown at 30°C (C and E) or 37°C (D and F) and prepared for immunofluorescence. Images are phase contrast (left), and FtsZ immunofluorescence (right). Scale bars are 5 µm. (TIF) [file pgen.1002561.s001.tif]

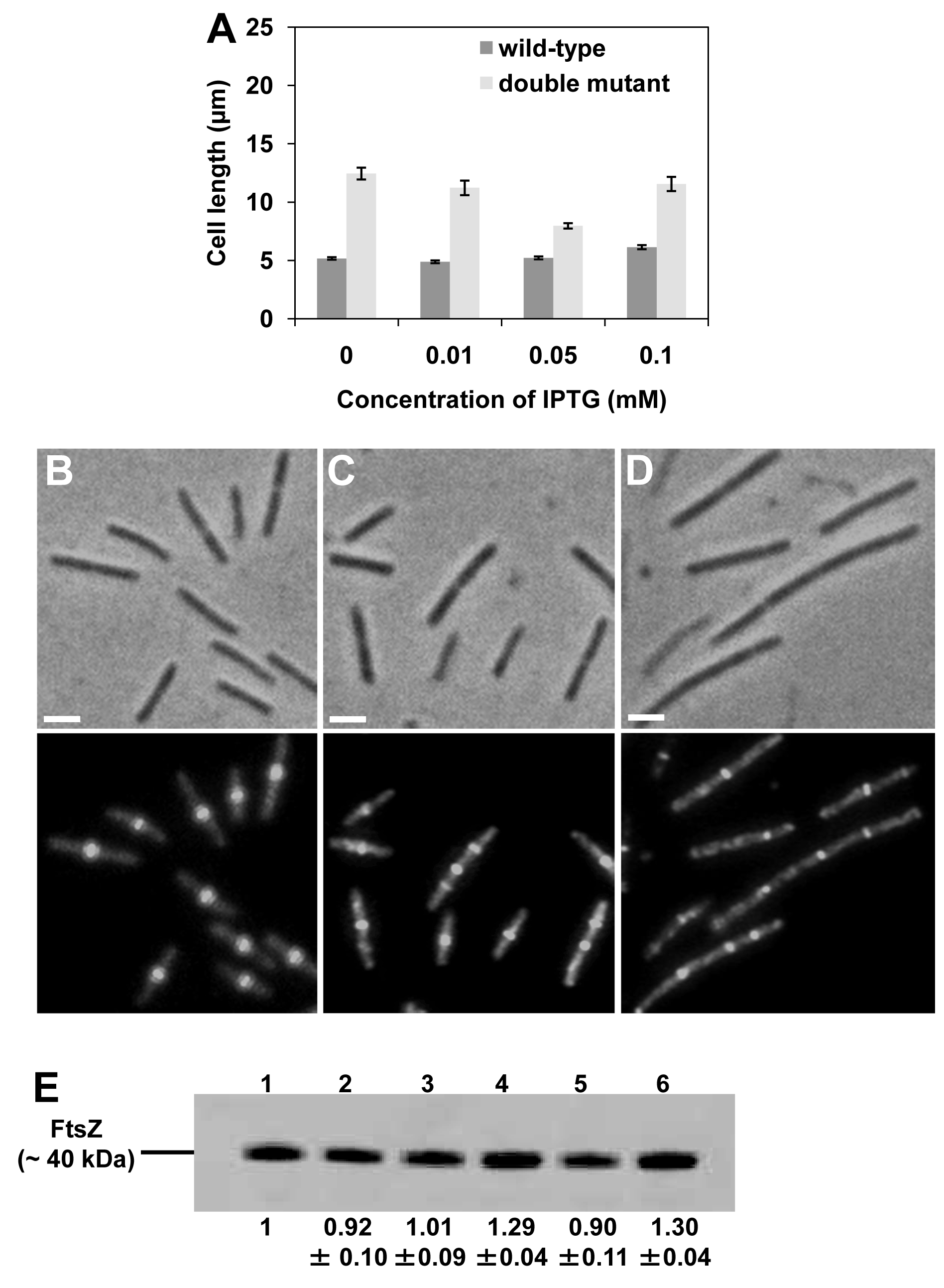

Supplement: Figure S2 — FtsZ overproduction in vegetatively-growing wild-type and noc minCD double-mutant cells grown at 30°C. Wild-type (SU558) and the double-mutant (SU685) cells containing Pspachy-ftsZ integrated at the amyE locus were grown in PAB at 30°C, with increasing concentrations of IPTG and collected during mid-exponential phase. (A) Cell length distribution of ethanol-fixed FtsZ-overproducing cells of the wild-type (dark grey bars) and the double-mutant (light grey bars) strains at 30°C. Error bars are µm ± SEM. (B and C) FtsZ localization in the wild-type grown in the absence (B) or presence of 0.05 mM IPTG (C). (D) FtsZ localization in the double-mutant strain grown in the presence of IPTG (0.05 mM). Images are phase contrast (upper panel), and FtsZ immunofluorescence (lower panel). Scale bars are 2 µm. (E) Western analysis of FtsZ levels in wild-type and double-mutant vegetatively-growing cells overproducing FtsZ at 30°C. Lanes were loaded with whole cell lysates of exponentially-growing cells including: (1) the wild-type strain not containing Pspachy-ftsZ (SU5) as a control; (2) the double-mutant strain not containing Pspachy-ftsZ (SU681); (3) the wild-type strain containing Pspachy-ftsZ, in the absence of IPTG (SU558); (4) the wild-type strain containing Pspachy-ftsZ, in the presence of 0.05 mM IPTG (SU558); (5) the double-mutant strain containing Pspachy-ftsZ, in the absence of IPTG (SU685) and (6) the double-mutant strain containing Pspachy-ftsZ, in the presence of 0.05 mM IPTG (SU685). Estimated molecular mass of the FtsZ band is 40 kDa. Numerical values under the bands show the mean (± standard deviation) intensity of the bands relative to the wild-type strain not containing Pspachy-ftsZ (SU5) (n = 2). FtsZ overproduction with 0.05 mM IPTG had very little effect on cell length in a wild-type background (SU558, Pspachy-ftsZ) at 30°C. Occasionally minicells (data not shown) as well as a slightly higher proportion of cells containing more than one Z ring (compare Figure [file pgen.1002561.s002.tif]

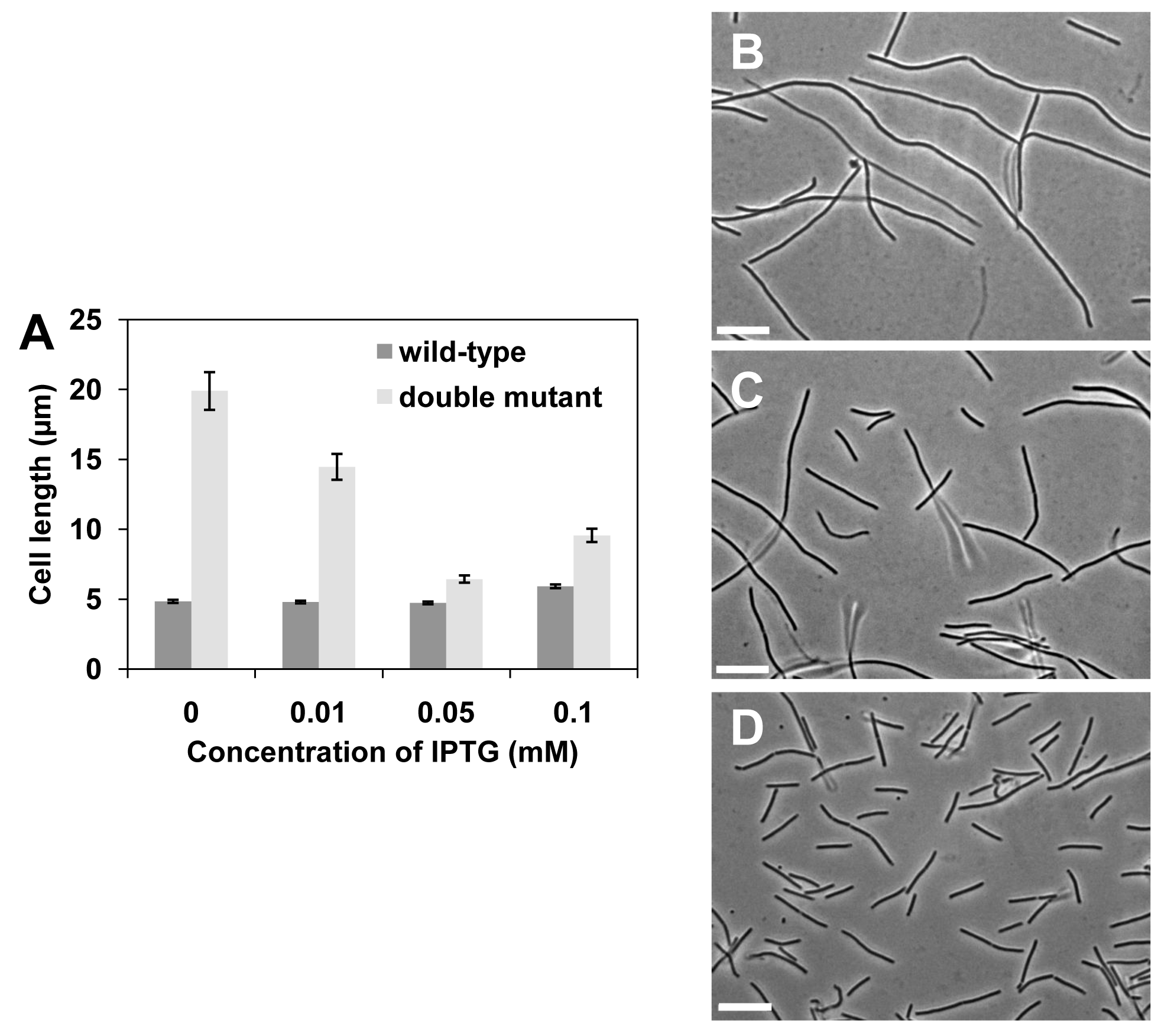

Supplement: Figure S3 — FtsZ overproduction in vegetatively-growing wild-type cells and noc minCD double-mutant cells at 37°C. Wild-type (SU558) and the double-mutant (SU685) cells containing Pspachy-ftsZ integrated at the amyE locus were grown in PAB at 37°C, with increasing concentrations of IPTG, collected during mid-exponential phase and fixed with ethanol. (A) Cell length distribution of FtsZ-overproducing cells of the wild-type (dark grey bars) and the double-mutant (light grey bars) at 37°C. Error bars are µm ± SEM. (B to D) Representative phase-contrast images of double mutant cells grown in absence of IPTG (B), with 0.01 mM IPTG (C) or with 0.05 mM IPTG (D). Scale bars are 5 µm. FtsZ overproduction with 0.05 mM IPTG had very little effect on cell length in a wild-type background (SU558, Pspachy-ftsZ) at 37°C. In the wild-type (SU558) and double-mutant (SU685) strain, growth in PAB at 37°C supplemented with 0.1 mM IPTG resulted in a detrimental effect on cell length (Figure S3A) and growth rate (judged by growth curve; data not shown). (TIF) [file pgen.1002561.s003.tif]

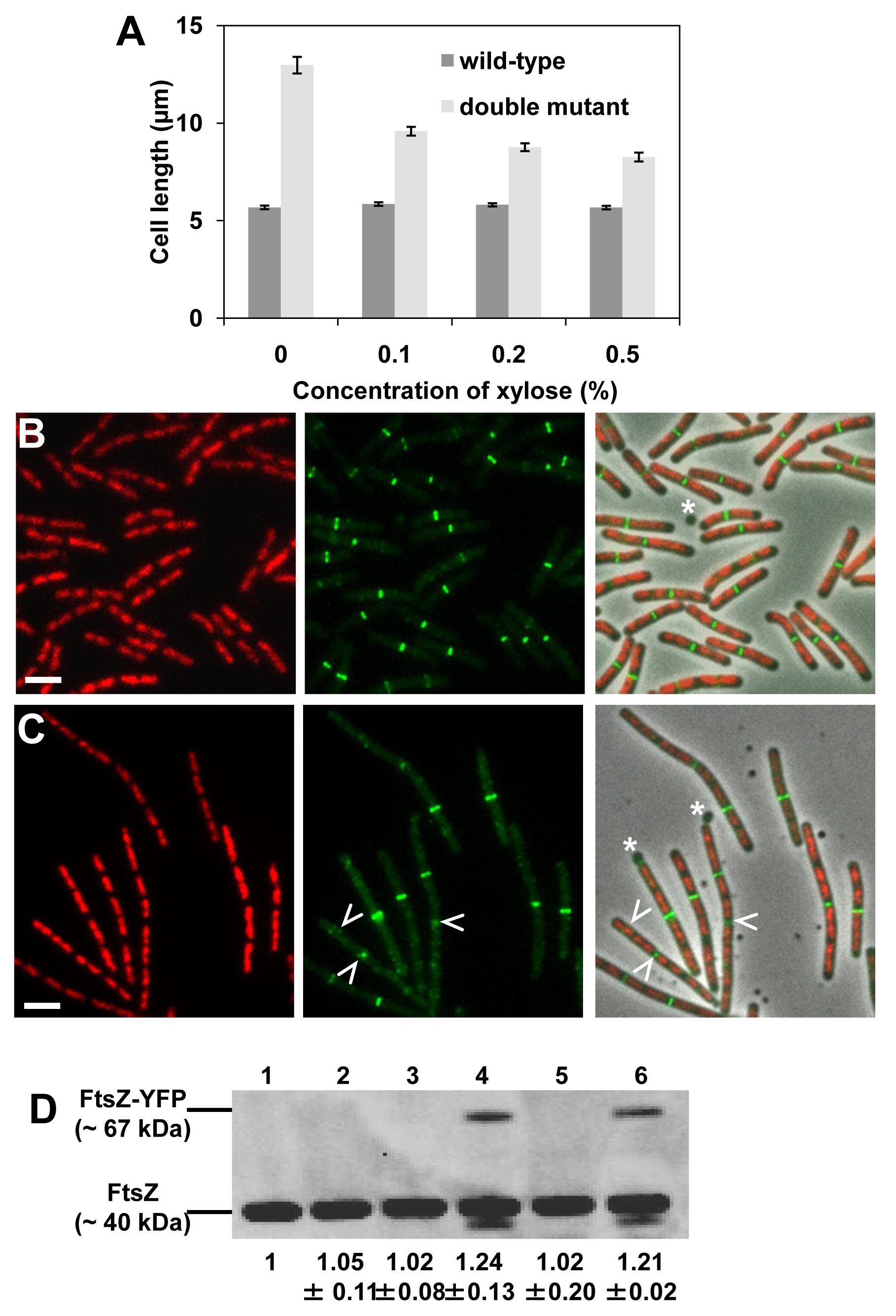

Supplement: Figure S4 — Overproduction of FtsZ using FtsZ-YFP in live wild-type and noc minCD double-mutant cells. Cells of the wild-type (SU492) and the double-mutant (SU663) strain containing Pxyl-ftsZ-yfp at the amyE locus were grown in PAB at 30°C, with various concentrations of xylose. Samples were collected during mid-exponential phase. (A) Cell lengths in ethanol-fixed wild-type (dark grey bars) and the double-mutant (light grey bars) cells during overproduction of FtsZ-YFP with increasing concentrations of xylose. Error bars are µm ± SEM. (B and C) FtsZ-YFP localization in live wild-type (B) and double-mutant cells (C) overproducing FtsZ using FtsZ-YFP (0.5% xylose) at 30°C. Images (left to right) are DAPI pseudo-coloured in red, FtsZ-YFP pseudo-coloured in green, and phase-contrast fluorescence overlay. Scale bars are 2 µm. Carets point to accumulations of FtsZ and stars denote minicells. FtsZ overproduction with 0.5% xylose had very little effect on cell length in a wild-type background (SU492). Occasionally minicells were observed. (D) Western analysis of total cellular FtsZ levels (both FtsZ-YFP and native FtsZ) in the double-mutant and the wild-type vegetatively-growing cells grown in the absence or presence of xylose (0.5%). Lanes contain: (1) the wild-type strain not containing Pxyl-ftsZ-yfp (SU5) as a control; (2) the double-mutant strain not containing Pxyl-ftsZ-yfp (SU681); (3) SU492 (Pxyl-ftsZ-yfp) with no xylose; (4) SU492 (Pxyl-ftsZ-yfp) with 0.5% xylose; (5) SU663 (minCD noc Pxyl-ftsZ-yfp) in the absence of xylose and (6) SU663 (minCD noc Pxyl-ftsZ-yfp) with 0.5% xylose. Estimated molecular mass of the bands is 40 kDa and 67 kDa, for FtsZ and FtsZ-YFP respectively. Numerical values under the bands shows the mean (± standard deviation) intensity of the FtsZ-containing bands relative to the wild-type strain not containing Pxyl-ftsZ-yfp (SU5) (n = 2). (TIF) [file pgen.1002561.s004.tif]

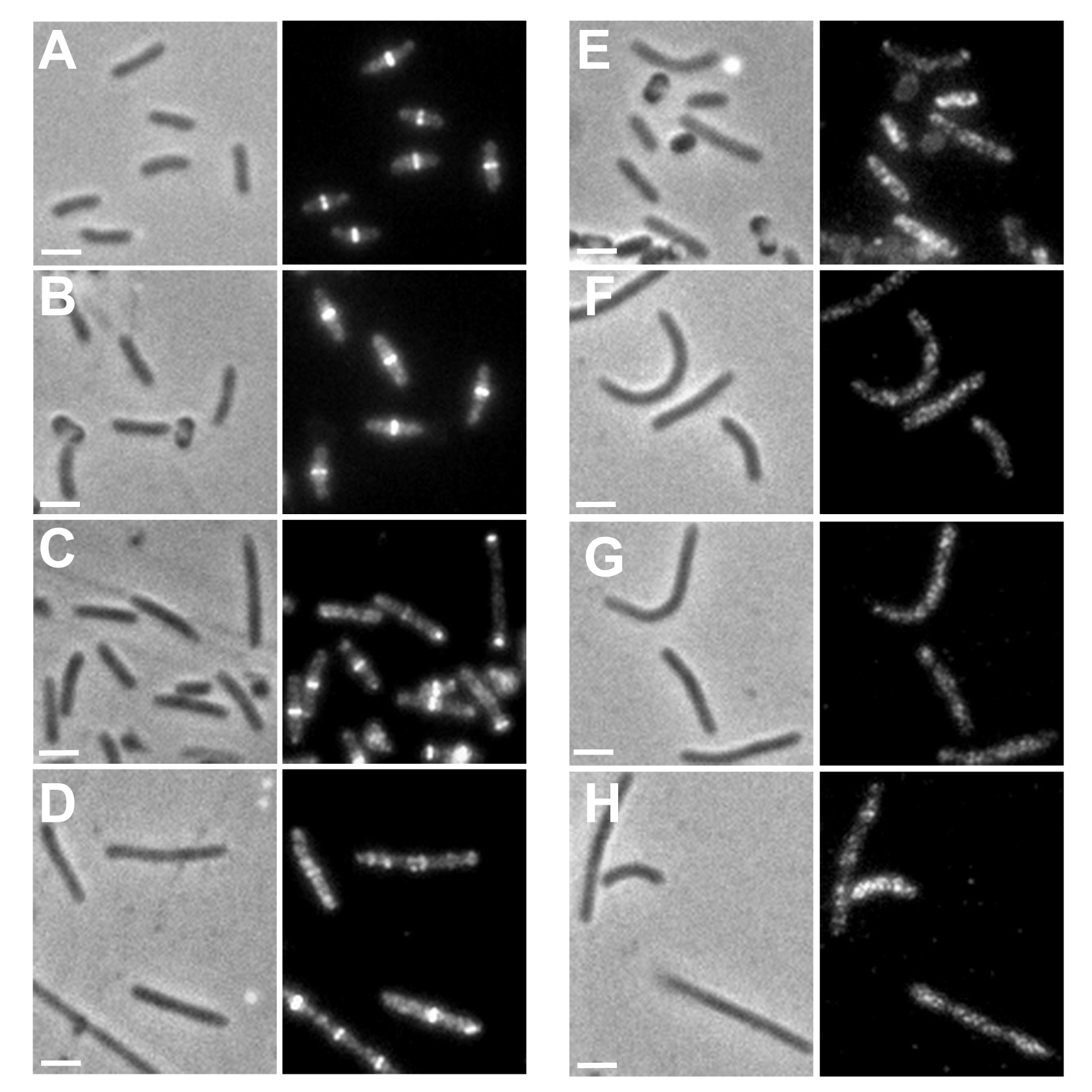

Supplement: Figure S5 — Z ring formation during spore outgrowth in dnaB (ts) cells is dependent on the addition of IPTG (and xylose) to the medium. Spores of dnaB (ts) strains containing Pspac-ftsZ integrated at the ftsZ locus and Pxyl-ftsZ-yfp at the amyE locus [SU671 (Min+, Noc+) SU678 (Min−), SU680 (Min−, Noc−) and SU683 (Noc−)] were outgrown at 34°C in PAB with (A to D) or without (E to H) 0.01% xylose and 1 mM IPTG, and collected for immunofluorescence at 150 min of spore outgrowth. Representative images of cells when Noc and MinCD are present (SU671) (A and E), when Noc is absent (SU683) (B and F), when MinCD is absent (SU678) (C and G) and when both Noc and MinCD are absent (SU680) (D and H). Images are phase contrast (left), and FtsZ immunofluorescence (right). Scale bars are 2 µm. (TIF) [file pgen.1002561.s005.tif]

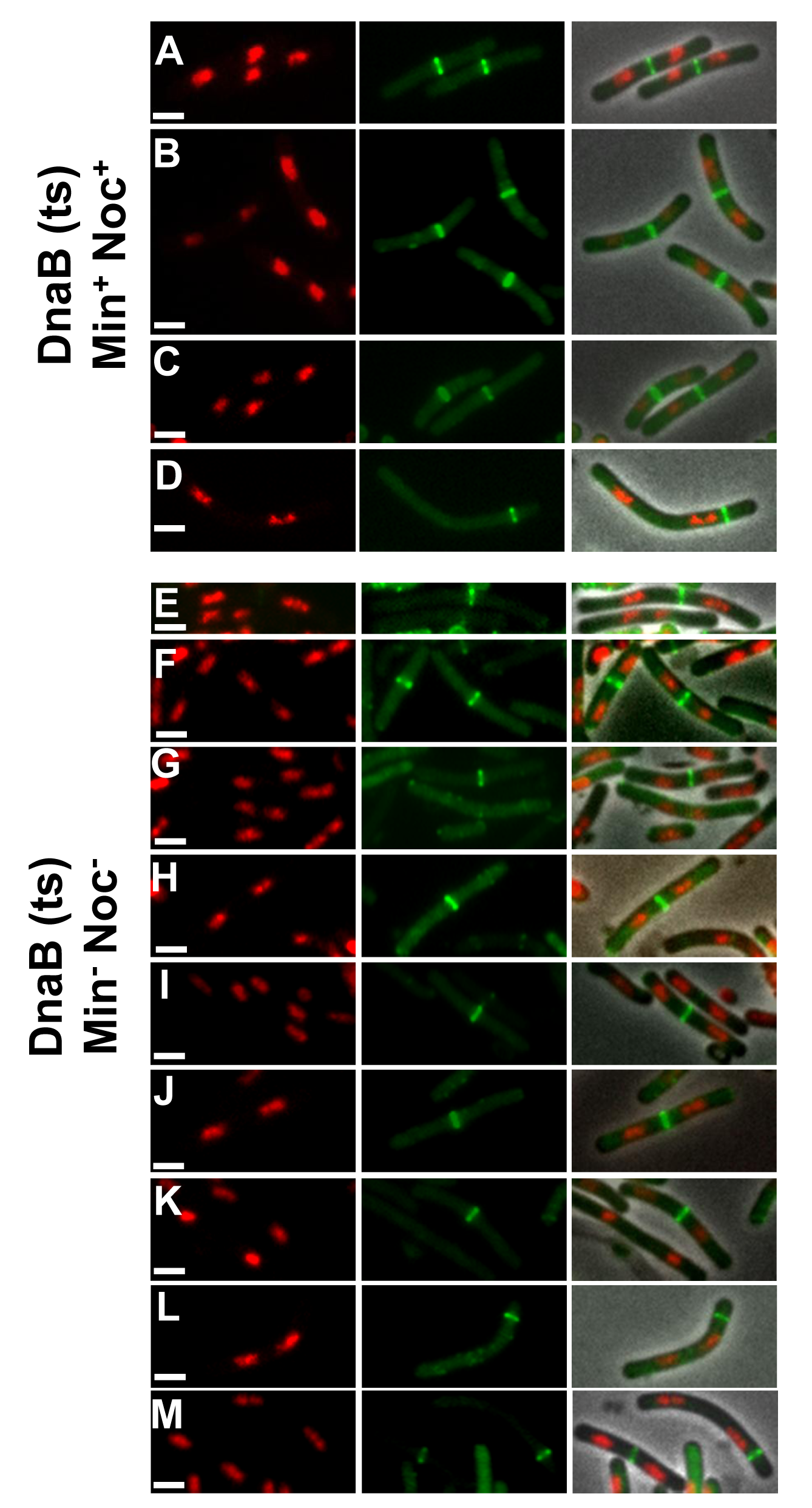

Supplement: Figure S6 — Z ring formation and positioning during spore outgrown in dnaB (ts) cells with two separated nucleoids that contain or lack both MinCD and Noc. See Figure 4A, for diagram, of experimental approach. Spores of the dnaB (ts) strains containing ftsZ::Pspac-ftsZ and amyE::Pxyl-ftsZ-yfp [SU671 (Min+, Noc+), SU680 (Min−, Noc−)] were germinated as described in the legend of Figure 4A. (A to D) Z ring localization in live cells with two nucleoids in cells containing both Noc and MinCD (SU671). (E to M) Z ring localization in live cells with two nucleoids in cells lacking both Noc and MinCD (SU680). (A to C and E to K) Z rings localizing between the two nucleoids. (D, L and M) Z rings localizing between the nucleoid and pole. Images (left to right) are DAPI pseudo-coloured in red, FtsZ-YFP pseudo-coloured in green and phase-contrast fluorescence overlay. Scale bars are 2 µm. (TIF) [file pgen.1002561.s006.tif]

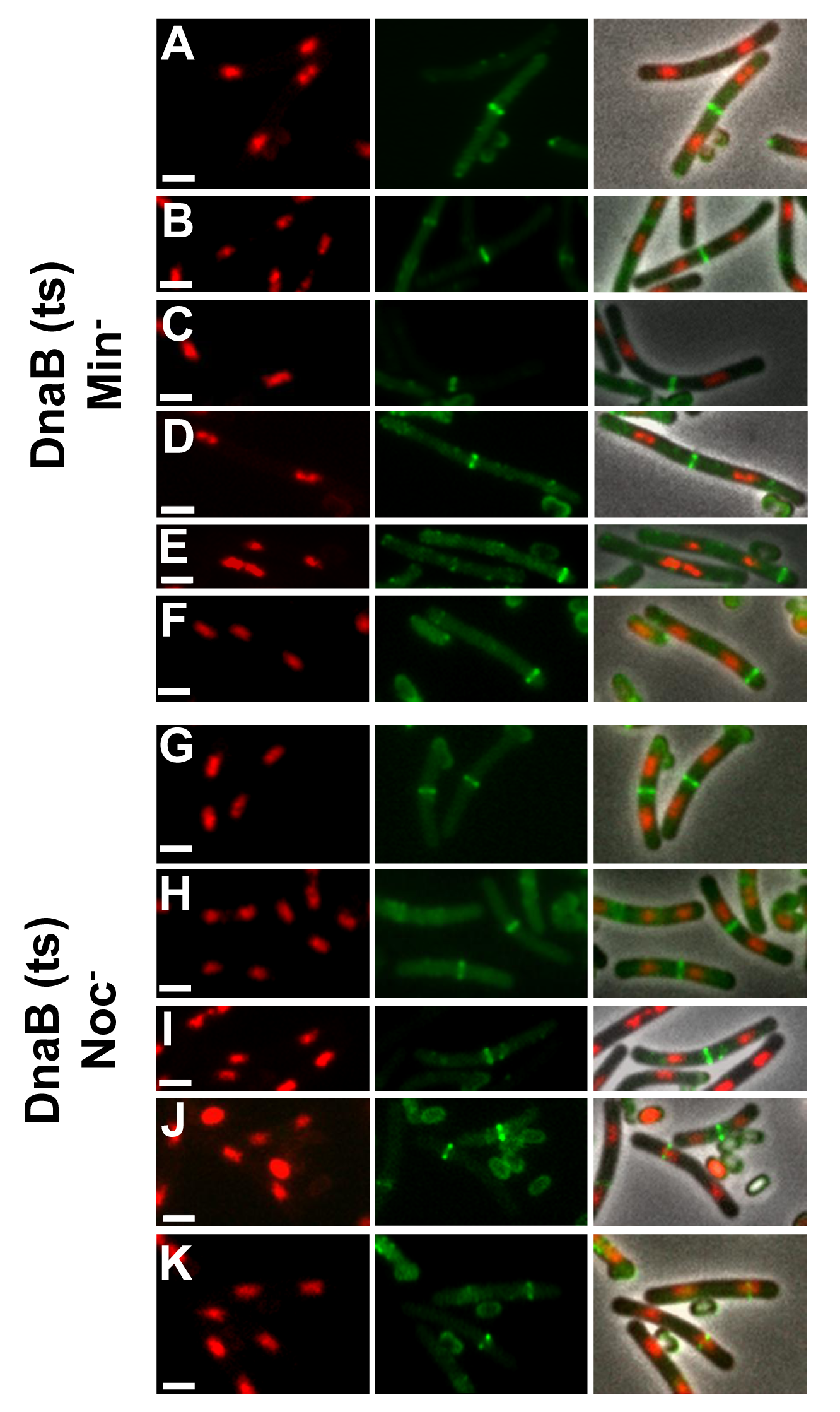

Supplement: Figure S7 — Z ring formation and positioning during spore outgrown in dnaB (ts) cells with two separated nucleoids that lack either MinCD or Noc. See Figure 4A, for diagram, of experimental approach. Spores of the dnaB (ts) strains containing ftsZ::Pspac-ftsZ and amyE::Pxyl-ftsZ-yfp [SU678 (Min−), SU680, SU683 (Noc−)] were germinated as described in the legend of Figure 4A. (A to F) Z ring localization in live cells with two separated nucleoids lacking MinCD. (G to K) Z ring localization in live cells with two separated nucleoids lacking Noc. (A to D and G to I) Z rings localizing between the two nucleoids. (E and F) Z rings localizing between the nucleoid and pole. (J and K) Z rings localizing between the two nucleoids (J) and Z rings localizing over one of the nucleoids (K). Images (left to right) are DAPI pseudo-coloured in red, FtsZ-YFP pseudo-coloured in green and phase-contrast fluorescence overlay. Scale bars are 2 µm. (TIF) [file pgen.1002561.s007.tif]

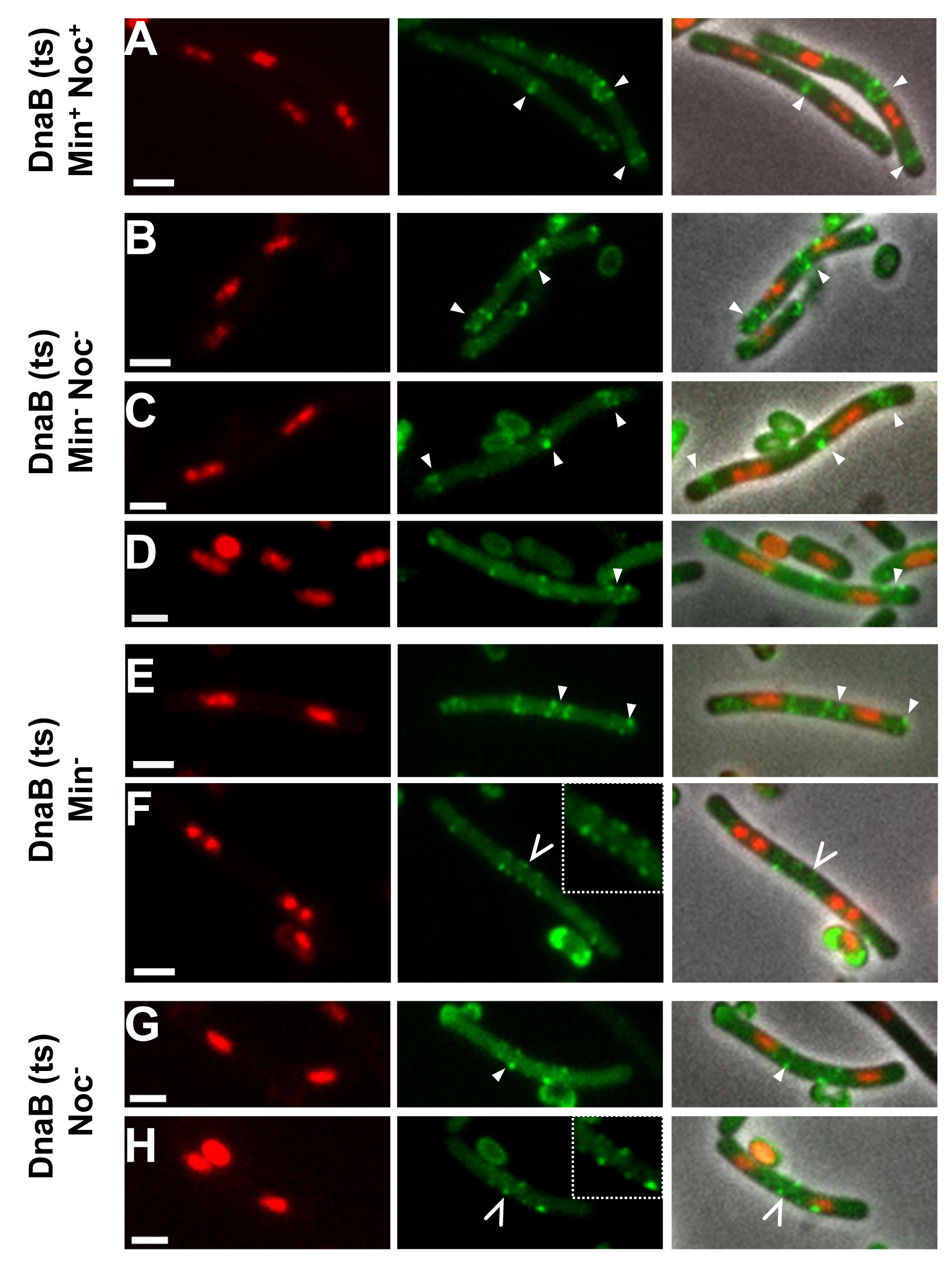

Supplement: Figure S8 — Distinct accumulations of FtsZ in outgrown dnaB (ts) cells with two separated nucleoids. See Figure 4A, for diagram, of experimental approach. Spores of the dnaB (ts) strains containing ftsZ::Pspac-ftsZ and amyE::Pxyl-ftsZ-yfp [SU671 (Min+, Noc+) SU678 (Min−), SU680 (Min−, Noc−) and SU683 (Noc−)] were germinated as described in the legend of Figure 4A. (A to H) FtsZ localizations in cells containing both MinCD and Noc (A), in cells lacking both MinCD and Noc (B to D), in cells lacking MinCD (E and F), and cells lacking Noc (G and H). Images are DAPI pseudo-coloured in red (left), FtsZ-YFP pseudo-coloured in green (middle) and phase-contrast overlay (right). Carets point to likely helical-like patterns which are magnified in (F) and (H). Small, white arrowheads point to distinct foci or other less distinct accumulations of FtsZ. Scale bars are 2 µm. (TIF) [file pgen.1002561.s008.tif]
